# A Mixed Methods Study of a Health Worker Training Intervention to Increase Syndromic Referral for *Gambiense* Human African Trypanosomiasis in South Sudan

**DOI:** 10.1371/journal.pntd.0002742

**Published:** 2014-03-20

**Authors:** Jennifer J. Palmer, Elizeous I. Surur, Francesco Checchi, Fayaz Ahmad, Franklin Kweku Ackom, Christopher J. M. Whitty

**Affiliations:** 1 Faculty of Infectious and Tropical Diseases, London School of Hygiene and Tropical Medicine, London, United Kingdom; 2 Medical Emergency Relief International, Nimule, South Sudan; 3 Islamic Relief, Islamabad, Pakistan; 4 International Medical Corps, London, United Kingdom; Makerere University, Uganda

## Abstract

**Background:**

Active screening by mobile teams is considered the most effective method for detecting *gambiense*-type human African trypanosomiasis (HAT) but constrained funding in many post-conflict countries limits this approach. Non-specialist health care workers (HCWs) in peripheral health facilities could be trained to identify potential cases for testing based on symptoms. We tested a training intervention for HCWs in peripheral facilities in Nimule, South Sudan to increase knowledge of HAT symptomatology and the rate of syndromic referrals to a central screening and treatment centre.

**Methodology/Principal Findings:**

We trained 108 HCWs from 61/74 of the public, private and military peripheral health facilities in the county during six one-day workshops and assessed behaviour change using quantitative and qualitative methods. In four months prior to training, only 2/562 people passively screened for HAT were referred from a peripheral HCW (0 cases detected) compared to 13/352 (2 cases detected) in the four months after, a 6.5-fold increase in the referral rate observed by the hospital. Modest increases in absolute referrals received, however, concealed higher levels of referral activity in the periphery. HCWs in 71.4% of facilities followed-up had made referrals, incorporating new and pre-existing ideas about HAT case detection into referral practice. HCW knowledge scores of HAT symptoms improved across all demographic sub-groups. Of 71 HAT referrals made, two-thirds were from new referrers. Only 11 patients completed the referral, largely because of difficulties patients in remote areas faced accessing transportation.

**Conclusions/Significance:**

The training increased knowledge and this led to more widespread appropriate HAT referrals from a low base. Many referrals were not completed, however. Increasing access to screening and/or diagnostic tests in the periphery will be needed for greater impact on case-detection in this context. These data suggest it may be possible for peripheral HCWs to target the use of rapid diagnostic tests for HAT.

## Author Summary

Human African trypanosomiasis (HAT or sleeping sickness) is a fatal but treatable disease affecting poor people in sub-Saharan Africa. Most HAT diagnostic equipment, infrastructure and expertise is located in hospitals. The expense of expanding testing services to remote areas using mobile teams severely restricts their use. Non-specialist healthcare workers (HCWs) in first-line (primary) health care facilities can contribute to control by identifying patients in need of testing based on their symptoms. We therefore trained first-line HCWs to recognise potential syndromic cases of HAT and refer them to a hospital screening service. Against a low baseline of HCW HAT referral experience, four months after the intervention, HCW knowledge of HAT symptoms increased and HCWs in 71.4% of facilities across the county had made referrals, incorporating new and pre-existing ideas about HAT case detection into referral practice. There was only a modest increase in numbers of referred patients received at the hospital for screening, however, largely because of distance. In an era where approaches to HAT case detection and control must increasingly be integrated into health referral systems, it is vital to understand the opportunities and challenges associated with syndromic case detection in first line facilities to design effective interventions.

## Introduction

Found in remote sub-Saharan areas where health systems are often weak and/or destabilised by armed conflict, human African trypanosomiasis (HAT, or sleeping sickness) is one of the world's most neglected tropical diseases (NTDs). It is caused by infection with trypanosome parasites that are transmitted primarily by tsetse flies (*Glossina*) and is nearly always fatal if untreated. HAT caused by *Trypanosoma brucei gambiense* represents more than 90% of global HAT burden and is endemic in geographically limited foci in west and central Africa [1]. In these areas, humans are assumed to be the main reservoir of infection.

HAT can be asymptomatic or involve non-specific symptoms in the first stage of disease. Characteristic symptoms involving mental and physical deterioration progressing to death are more likely to appear in the second stage, once parasites have entered the brain [2], [3]. The natural duration of *gambiense* HAT is thought to be a median of almost a year and a half for each stage [4].

Systematic active screening (AS) and treatment of at-risk populations using laboratory-equipped mobile teams identifies both symptomatic and non-symptomatic cases and has been a key method used to control recent large epidemics in central Africa [1], [5]. It is also, however, resource-intensive and, for this reason, has not been sustained in many areas as caseloads have decreased [5]–[8], despite calls for an intensification of control activities to achieve elimination [9]. This has been especially problematic in South Sudan where, since the signing of the north-south peace agreement in 2005, the country has seen a critical decrease in funding for HAT control activities as international non-governmental organisations have disengaged from HAT service delivery [7]. Approaches to screening which are sustainable at low incidence rates and low funding rates will need to be used to maintain case detection and control activities in this type of context. Building these around existing health services is likely to be the most pragmatic and sustainable approach.

One option that has been tried in the past is to split the work involved in active case-finding into parts, using community-based healthcare workers (HCWs) to collect blood samples on foot for subsequent screening at hospital laboratories, where most HAT diagnostic equipment and expertise is located [10]–[13]. Newly-introduced rapid diagnostic tests (RDTs) for HAT could similarly be used [14]. Fewer resources can then theoretically be spent on following-up a smaller number of people for confirmatory testing.

Another option is systematically to screen populations when they come into contact with health facilities where screening resources are already available [13], [15]–[17], either comprehensively or under programmes targeting specific populations as has been suggested for antenatal care [18]. Targeting of children at routine child health visits or via school health programmes is not typically prioritised since adults are more likely to be infected [19]. Currently, neither of these approaches to case-detection is frequently used.

**Figure 1:**
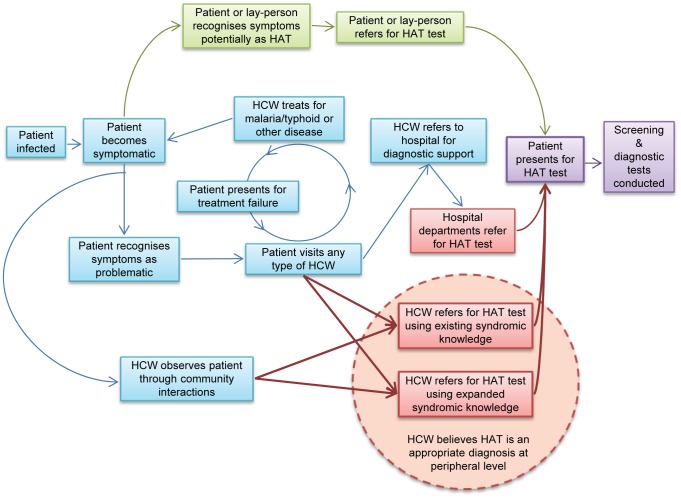
Treatment-seeking and test referral pathways which lead to successful HAT detection via passive screening. Boxes and arrows in green represent HAT test referral because the patient or a lay-person suspects symptoms may be due to HAT. Boxes and arrows in red represent HAT test referral because a HCW suspects symptoms may be due to HAT. Thick red arrows are the pathways targeted by the HCW HAT training intervention, which are influenced by HCW perceptions of the appropriateness of HAT referral at the peripheral level. Boxes and arrows in blue and purple represent treatment-seeking events that precede and follow consideration of HAT referral, respectively.

A third cost-effective alternative is to target screening resources to symptomatic patients who have a higher probability of being HAT cases than the general population. This is the principle behind referral-based passive screening (PS), the least resource-intensive and most commonly used alternative to AS. Under this approach, patients may be referred for HAT screening by a HCW or by a lay-person (family member, neighbour, church leader, etc) knowledgeable about HAT symptoms and HAT test availability (Figure 1). Recognition of symptoms to prompt HAT referral, however, is commonly thought to be delayed by lengthy and/or complicated preceding treatment-seeking events including syndromic misdiagnosis of malaria, typhoid or HIV [20]–[24]. The specificity of RDT- and microscopy-based diagnosis for malaria, HIV and HAT are also each lowered in the presence of co-infections [25]–[27]. In a context of extreme material poverty, as in most highly HAT-endemic areas, transportation, opportunity and direct costs of recurrent treatment-seeking may severely diminish patient motivation to keep seeking treatment or complete referrals [22], [28], [29]. HAT referral completion may also potentially be influenced by patient perceptions of severity and treatability of symptoms [22], knowledge of treatment requirements or associated cultural prohibitions [30], [31], or HAT-related stigma [30].

While PS is often the approach responsible for detecting the majority of HAT cases over the length of a program [21], [22], [32]–[35], providing much of the information on incidence used to quantify the burden of HAT locally and globally, surprisingly little attention is paid to optimising its use. Syndromic algorithms that can be used to guide HCW referral have recently been proposed by us [36] but most hospital programmes employ no systematic approach to identify potential cases. Additionally, outside of a few outbreak situations when AS is not feasible [37], [38], typically little emphasis is placed on education or sensitisation of HCWs about the signs and symptoms of HAT which should trigger referral. Furthermore, the contribution that HCWs in peripheral facilities could make to syndromic case detection is habitually over-looked in vertically-organised HAT control programmes [37], [39].

One notable exception to this trend was a five year programme in Niaki focus, Republic of Congo, in the 1980s, which trained HCWs in all first-level public facilities to screen syndromically and detect cases via basic microscopy [15]. Patients could also be syndromically referred for serological and full confirmatory screening at a central hospital. This strategy appears to have greatly improved participation of peripheral facilities in case-detection. By its final year, these facilities were diagnosing more new cases from rural areas than the referral hospital or mobile teams (and responsible for >30% of the total). A simpler integrated case detection strategy based on syndromic detection in peripheral facilities has recently been suggested for the Democratic Republic of Congo [22]. Under it, referrals could be made to a central facility or specialised supervisors could perform screening tests on visits. The deployment of RDTs for HAT to first or second-line facilities in a passive screening approach could also incorporate syndromic referral rationale to conserve test resources. Others have called for specific syndromic training on HAT for peripheral HCWs [20], [24], [37] but, to our knowledge, no such intervention has been conducted and systematically evaluated.

In this study, we present the results of a syndromic HAT training intervention which targeted HCWs in peripheral facilities in the Nimule focus of South Sudan with the objective of increasing the rate of syndromic referrals to a central screening and treatment centre.

## Methods

### Ethics statement

This study was approved by the London School of Hygiene & Tropical Medicine's ethical review committee and was registered with the Ministry of Health, Government (now Republic) of South Sudan. All HCW participants provided written informed consent and patients provided verbal informed consent. Verbal consent was approved by the LSHTM committee for use with patients because of low literacy rates in the study area. Receipt of consent was documented on data collection forms.

### Study setting

In South Sudan, training on HAT is offered through the national control programme by World Health Organisation staff but is currently targeted to hospital-based inpatient HCWs on the clinical management of patients and to laboratory staff on diagnostic protocols. HCWs of all levels who have received formal training in South Sudan are briefly introduced to HAT symptomatology, diagnosis and treatment in curriculum. Many informally trained HCWs who are nevertheless popular providers of patient care in this context have never received any training on syndromic HAT recognition.

HAT screening and diagnosis is currently only available at six hospitals in the country [7]. In the Nimule focus of Magwi County (MC), Eastern Equatoria State, HAT testing and treatment services are available at a single site, Nimule Hospital, supported by the non-governmental organisation, Merlin (Medical Emergency Relief International) [7]. Services in this historic focus were re-introduced at the end of the Sudanese civil war, in 2005. Transmission is thought to have increased in recent years due to population movements of internally-displaced and returning refugee populations from neighbouring endemic areas. Small-scale (<30% population coverage) AS surveys conducted in 2005, 2006 and 2008 revealed a low-to-moderate estimated HAT prevalence of around 1% with the highest prevalence in any village estimated at 5.8% [40]. No surveys have been conducted in 2/6 payams (districts) furthest from the hospital in the east of the county but cases have been identified from these areas via passive screening.

Preliminary formative research by us suggested a need to improve knowledge of HAT symptoms and screening service availability among Nimule area peripheral HCWs as a necessary (although possibly not sufficient) step to increase screening [40]. Excessive sleeping, mental confusion, ‘swollen’ body parts due to oedema or weight gain, pain, fever and enlarged lymph nodes appeared to be the key symptoms which signalled HAT to referring HCWs. Along with these symptoms, excluding common diagnoses such as malaria and typhoid was perceived by HCWs as a valuable strategy which helped them identify likely HAT cases. Few HCWs appeared routinely to make HAT referrals, however, either because making HAT diagnoses was perceived as something that should be done by hospital-based mobile teams or because they did not recognise the local magnitude of the problem (and hence would not consider HAT as a possible diagnosis) without the cue of a large community campaign.

### Overall study design

HCWs from peripheral healthcare facilities were trained to recognise potential syndromic cases of HAT during routine outpatient practice and refer them for screening to Nimule hospital. The rate of patient referrals received at the hospital from a peripheral healthcare facility for HAT screening before versus after the intervention was the primary measure of intervention effectiveness. Secondary (intermediate) measures were changes in the number and distribution of HAT referrals made by trained HCWs in the county and changes in HCW knowledge of HAT symptoms.

Based on interviews with peripheral HCWs about their existing HAT referral practices before the intervention, we theorised that the training intervention, if effective, could increase the rate of HAT referrals made in two main ways: by expanding the range of symptoms that prompt HCWs to make HAT referrals or by encouraging HCWs to think of HAT as an appropriate diagnosis to make at the peripheral level, or both (Figure 1). Common patient case-finding techniques and clinical reasons for referrals after the training were therefore identified qualitatively with HCWs to explore which type of syndromic knowledge best explained changes in referral volumes. The appropriateness of referrals made by peripheral HCWs was also appraised *a posteriori* by a panel of four clinicians with experience of HAT, based on reported signs and symptoms.

Finally, barriers that HCWs faced to support completion of referrals by patients were also explored.

Hospital-based data collection on pre- and post-intervention referrals took place over the 10 month period, July 2009–April 2010. HCW HAT training took place during the month of November 2009. Facility-level referral data were collected during a four-week evaluation period in March 2010.

### Intervention

This training intervention was designed according to three principles. First, it should achieve high coverage of HCWs in all types of provider setting from which patients with sleeping sickness might seek care. Second, it should be realistic in terms of what could be delivered by government or non-governmental organisation programmes at scale. Third, it should specifically recognise existing HAT referral strategies and discuss local barriers to syndromic case detection as identified from preliminary qualitative research ([40] and others).

The training was delivered through a series of six one-day workshops in each payam of the county. Invitations were distributed to facilities via local authorities and health organizations one month before the training. During briefing visits, local authorities were asked to identify the location of all public and military health facilities in the payam as well as any private facilities and traditional herbalists or witch doctors (henceforth referred to as traditional practitioners) they were aware of. Additional private facilities were added to the invitation list by driving or walking around payams and noting health structures. Chiefs and HCWs were also interviewed to identify popular local traditional practitioners. Facilities were invited to send as many HCW-diagnosticians to the workshops as could be spared without disrupting essential services. HCWs from small drug shops were invited if they reported that they examined and diagnosed patients on their premises. Nimule hospital HCWs were excluded from the HAT training workshops unless they also operated peripheral private facilities; outpatient staff had already been sensitised to HAT symptomatology during HAT patient management training earlier in the year. Participants were offered no training incentives other than lunch, a training manual and a certificate of attendance.

The training workshops were conducted by the Nimule hospital HAT programme manager (ES) and an expatriate HAT researcher (JP) in English and either of the two local languages, Madi or Acholi. Arabic simultaneous translations were also offered as needed. Training was didactic as well as participative and covered several topics: HAT transmission, distribution, control, clinical signs & symptoms, diagnosis and treatment, as well as a discussion on taking patient histories, recording referrals and counselling patients to complete referrals. No specific syndromic referral algorithm was introduced.

Key messages discussed about local barriers to syndromic HAT case-detection included: the problematic expectation among HCWs and lay people that HAT patients present more often with an increased rather than decreased appetite, the complication that frequent drunkenness adds to identifying HAT symptoms, the particular difficulty that imprisonment for mental symptoms poses for soldiers to seek treatment, and management of differential biomedical diagnoses that can be confused with HAT (especially malaria; magical poisoning and witchcraft were not discussed). The problematic history of local HAT programme funding was presented with the aim of empowering HCWs to play a greater role in case-detection outside of hospital-directed HAT activities (see File S1 for training manual). Workshops finished with a group memory game in which participants were asked to guess which HAT symptoms were being acted out by other participants.

### Evaluation

#### HCW questionnaires

Before the training session began, participants were asked to complete a written questionnaire (see File S2) that collected information on demographics, service availability at the facilities where they worked and baseline experience with referring patients for HAT. Questionnaires were written in English and any participants who could not read English completed the questionnaires through verbal translations. Unique identifier codes, rather than names, were used on questionnaires.

Questionnaire and knowledge test data were entered into EpiData (EpiData Association, Odense, Denmark 2000–2010) and double-checked for errors in entry by a second researcher, then exported to Stata 11 (StataCorp, Texas) and cleaned before analysis. Simple tabulations were made for questionnaire variables and demographic sub-groups were compared using chi-squared or Fisher's exact tests of proportions.

#### HAT knowledge tests

To assess the effectiveness of the training workshops in consolidating pre-existing knowledge and improving the range of correct signs and symptoms (hereafter referred to only as symptoms) that HCWs associated with HAT, we administered HAT symptom knowledge tests. Participants were asked to complete the tests at three time points: immediately before the workshop (pre-test), immediately after the workshop (post-training test) and 4 months after the workshop, during the evaluation period (post-intervention evaluation test). Participants were asked to associate 14 symptoms with HAT, by answering “yes”, “no” or “I don't know” to the question: Is this a sign or symptom of sleeping sickness?

Changes in overall knowledge were assessed using participants' mean test scores. Mean test scores for individual participants were computed only if they responded to all 14 symptom association questions in each HAT knowledge test. In instances when participants chose the response “I don't know” in relation to whether a symptom was associated with HAT, these were classified as incorrect under the assumption that participants who were not confident enough in their HAT knowledge to choose a “yes” or “no” response may similarly not be confident enough in their knowledge of HAT-specific symptoms to make HAT referrals. Overall changes in HAT knowledge between tests were assessed using Wilcoxon signed rank tests. Changes in correct associations of individual symptoms with HAT were explored using McNemar's chi-squared tests. As in analysis of HCW reasons for referral (see below), changes in individual symptom scores were also qualitatively assessed in terms of whether these were based on mainly new or pre-existing HAT case detection ideas. Kruskal-Wallis tests were used to test for differences in test scores across demographic variables (or responses to certain questionnaire items) at a given time point.

#### Peripheral facility referral monitoring

All facilities were asked to record prospectively HAT referrals made on forms distributed at the training and told they would be collected during evaluation visits. This data collection system could not be implemented before the intervention to provide a baseline, as it would have entailed training HCWs in HAT recognition and referrals ahead of the intervention itself. There was no general referrals recording system in place in facilities across the study area from which to estimate reliably baseline HAT referrals from. Instead, two self-reported baseline measures were collected in a written questionnaire. First, HCWs were asked to report whether they had ever in their life referred for HAT. Second, HCWs were asked retrospectively to estimate the number of HAT referrals they had made in the month before training. Participants were not asked to distinguish between ‘new’ (never been serologically suspected or treated for HAT) and ‘old’ HAT referrals made, as it was unlikely that this information could be verified. In all other analyses, information is reported for new patient referrals only.

HCWs were asked to record patient demographic details and report any HAT-specific attributes present at referral by choosing from a list of 32 symptoms, signs or epidemiological criteria based on their clinical investigations. Trained traditional practitioners and payam health authorities were asked to refer potential HAT cases to the nearest public or military health facility for recording. Previous qualitative research with traditional practitioners suggested that there were no traditional practices that could be used to treat HAT locally. HAT referral slips that patients could carry with them to the hospital were also provided to facilities.

By the evaluation period, patient referral forms were sometimes lost or inaccessible; additionally, forms were sometimes incomplete. In these instances the evaluation team recreated patient referral forms when possible by interviewing referring HCWs (patient demographic data on 32% of forms, symptom data on 40%).

#### Hospital-based referral monitoring

In addition to standard patient demographic and test outcome information, data on the source of referral was collected for all patients passively screened for HAT at Nimule hospital, Jul 2009–Apr 2010. Information on all HAT-specific symptoms in presenting patients was also collected Oct 2009–Apr 2010. All lab staff were trained in data recording 1–2 months before both types of data collection began, so that by the beginning of the study period this information was routinely collected. However, from January 2010, the hospital lab experienced a critical staff shortage, such that HAT screening was unavailable to self-referred patients for a 7 week period (Jan–Mar 2010) of the post-training study period.

Other information sources were systematically searched for patients potentially missing from the HAT screening register including inpatient lab request logs, medical inpatient ward logs, HAT treatment admission logs and individual HAT patient files. Blood donors routinely screened for HAT were excluded from all analyses. Screening data were double-entered into an EpiData database and transferred to Stata for cleaning and analysis.

#### Analysis of referral monitoring data

To calculate the peripherally-referred HAT patient screening rate before versus after the training intervention, the pre-intervention period was defined as 1 Jul–31 Oct 2009 (4 months) and the post-intervention period as 1 Dec 2009–31 Mar 2010 (4 months). Population estimates for the rate denominators were taken from Ministry of Health 2009 projections of 2008 census data, and published estimates of the IDP population in Magwi County [41].

For the purpose of computing detection rates and percent test positivity, a HAT case was defined as (i) positive microscopy on lymph fluid directly or on blood using hematocrit concentration (Woo test) or (ii) positive CATT on diluted blood serum at dilution 1∶16, as per Merlin HAT protocol.

#### Determination of referral appropriateness

The syndromic appropriateness of referrals made by peripheral HCWs after the training intervention was assessed using expert clinician review of symptoms recorded in peripheral facility referral logs according to methods already described [36]. The proportion that would have been referred according to a syndromic referral algorithm which was subsequently identified by us as appropriate for this context [36] (at least one of: sleep problems, neurological problems, weight loss or history of oedema) was also calculated.

#### Interviews on reasons for referral and referral non-completion

Evaluation interviews with referring HCWs also provided qualitative information on HAT referrals made after the intervention. For each referral, HCWs were asked to recall: (i) the circumstances of referral (how and where HCWs encountered symptomatic patients), (ii) the major syndromic and clinical considerations they used to make the referral, and, (iii) where HCWs could comment, information on the outcome of the referral (whether the referral was completed and reasons for non-completion, if known). Patients who had not yet presented to Nimule hospital for screening could not be systematically followed-up because of time constraints.

In retrospectively exploring HCWs' reasons for referral, the central analytic task was to identify which symptoms and narratives about how to find HAT cases were commonly being operationalised in HCW explanations of referrals after the training, taken to be reflective of actual practice which could not otherwise be observed. Reasons given in response to the open-ended question, “why did you think this person could have sleeping sickness?” were noted and HCWs were questioned about any additional symptoms recorded in referral logs. Symptoms and narratives could either be attributed to ideas about HAT case-detection which, according to preliminary qualitative research, mainly pre-existed before the training or to new ideas which were mainly introduced during the training, as theorised by the research team before the training intervention. Other referral ideas discussed in interviews were also noted.

#### Focus group discussions with HCWs

During evaluation visits, a subset of referring HCWs were asked to participate in a group discussion to provide additional contextual information on experiences with syndromic case identification, decision-making for referral and patient referral completion. Participants were invited from geographically clustered public and private facilities in 4/6 payams where the study was conducted (one in the hospital payam, one in a neighbouring payam and two in the most distant payams). Groups ranged in size from 3 to 6 participants. Discussions were held in English with simultaneous translations into Madi or Acholi for a minority of participants in two groups.

Group discussions and interviews with referring HCWs followed topic guides. Discussions in military barracks were held separately with referring HCWs and military commanders. After each set of facility interviews or group discussion, field notes were discussed by the research team to iteratively identify emerging themes and new lines of enquiry. Field notes and transcripts from interview and discussion recordings were imported into NVivo 8 qualitative analysis software (QSR International, Melbourne, Australia) for coding and thematic analysis by a single researcher (JP). Transcripts and notes from interviews with each HCW or group discussion were read in their entirety before coding line-by-line to identify and label ideas and meanings conveyed in each small section of text. These codes were then grouped and labelled to reflect broader themes. Further additions and revisions to the coding framework were made on a continual basis as higher level constructs were generated, through reviewing emerging themes and interpreting them in relation to the research objectives. Care was taken to explore any differences in coding between HCWs from different facility types and from different regions/ethnicities. Minor corrections to grammar were made in some interview excerpts presented here in order to improve their readability.

## Results

### Profile of HCWs and facilities that received HAT training

We trained 108 HCWs from 61 public, private and military health facilities, as well as 12 traditional practitioners and 3 local government health authorities (123 HCWs total, Table 1). Participants came from almost all (97.4%) public facilities in the county, all military facilities and from more than half (62.5%) of private facilities (Table 2).

**Table 1:** Characteristics of participants who received HAT training.

Characteristic	n*	%	Characteristic	n	%
Gender			Literacy		
Male	102	82.9	Literate	104	89.7
Age group			Facility type		
0–29 yrs	50	45.1	Public PHCU/PHCC	84	68.3
30–49 yrs	51	46.0	Private†	15	12.2
≥50 yrs	10	9.0	Military	9	7.3
Nationality			Traditional practitioner	12	9.8
Sudanese	111	98.2	Local government health dept	3	2.4
Other	2	1.8	Type of training		
Tribe			A: Clinical officer	6	4.9
Madi	38	33.9	A: Certificated nurse	7	5.7
Acholi	61	54.5	A: Enrolled nurse	4	3.3
Dinka	2	1.8	B: Community health worker	51	41.5
Other	11	9.8	C: Nursing assistant	25	20.3
Location (payam)			C: Lab technician	1	0.8
Nimule	16	13.0	C: Lab assistant	3	2.4
Pageri	26	21.1	C: Pharmacy assistant	1	0.8
Mugali	10	8.1	C: Community midwife	6	4.9
Magwi	25	20.3	C: Traditional practitioner	12	9.8
Pajok	21	17.1	C: No formal training	7	5.7
Lobone	25	20.3	No. patients seen/day (self-reported average)		
No. years working in study area			0–5 pts	26	23.6
<5 yrs	64	61.0	6–10 pts	12	10.9
5–9.9 yrs	19	18.1	11–15 pts	6	5.5
≥10 yrs	22	21.0	16–20 pts	17	15.5
			≥21 pts	49	44.6

*Totals do not always sum to 123, as questionnaire data were missing for several participants who attended training and only some items could be completed on their behalf from administrative records.

† 5 participants worked at both public and private facilities, but were counted as public facility employees only for this analysis.

**Table 2:** Numbers and types of peripheral health facilities represented at HAT training and followed-up during the evaluation period.

Facility type	N in county	N received HAT training	N visited during evaluation
Public PHCC	8	7	7
Public PHCU	30	30	29
Military	4	4	3
Private	32	20	10
Total facilities	74	61	49

PHCU = Primary health care unit, the lowest level of healthcare available to communities.

PHCC = Primary health care centre, the next highest level of care.

Participants were predominantly male and from the two indigenous tribes of the county: Madi and Acholi (Table 1). 13% of HCWs came from the payam where the hospital is located (Nimule), 29.2% from neighbouring payams (Pageri and Mugali) and 57.7% from payams distant from the hospital. Most HCWs were recently returned refugees (median number of years worked in the county: 3, range 0–42, 55% of participants >30 years of age) and had a low level of formal clinical education. Clinical officers (CO), who receive 3–4 years of clinical training, certificated nurses who receive three and enrolled nurses who receive two, made up only 13.9% of participants (training category A). Community health workers (CHWs) who receive nine months of clinical training made up 41.5% (category B) and participants working in health facilities with no formal clinical training (mostly nursing assistants trained on the job; category C) made-up the largest proportion (44.7%). HCWs who attended HAT training collectively estimated to see between 1400–1900 patients/day, equivalent to approximately 1% of the county population. As a group, CHWs engaged in the majority of patient consultations (462–550/day), followed by informally trained HCWs (378–450/day) and COs/nurses (189–225/day). Public health facilities were responsible for more consultations (861–1025/day) than private facilities (25–125/day), military facilities (25–75/day) and traditional practitioners (22–30/day); however, the under-representation of private facilities and traditional practitioners in this training workshop should be noted.

Private clinics were most likely to have any testing service available (78.6% vs 58.8% in public and 66.7% in military facilities, data not shown) and public facilities most likely to have only malaria rapid-diagnostic tests (RDTs) available. One facility (a private clinic) reported having the capacity to diagnose HAT microscopically. Stock-out of tests, reagents and equipment were, however, a problem in all but 12 facilities, and stock-out of drugs was a common reported reason why HCWs made referrals. Infectious diseases were the most common reason (34.6%) for the last general referral participants made. Participants collectively ranked a list of common illnesses/conditions for which treatment is commonly sought by patients in MC according to how common they are in the county, in the following order: malaria, drunkenness, syphilis, typhoid, HIV, mental illness, tuberculosis and magical poisoning, with HAT ranked last (only 9.9% rated HAT as “very common”; 63.1% rated HAT as “not common”).

#### Pre-intervention HAT referrals

Despite this ranking, 79 (69.6%) HCWs reported that they had seen someone with HAT before; just over a quarter (30 or 26.5%) of participants had ever referred someone for a HAT test (baseline measure 1, Table 3). In the month before the training workshop, 14 (12.4%) HCWs reported that they had referred one or more people for a HAT test. Collectively, participants reported having made 21 (new or old) HAT referrals from 11 facilities in the month before training (baseline measure 2). Participants were more likely to have made HAT referrals from private (28.6%) and military (57.1%) than public facilities (7.7%) in the month before training, with traditional practitioners and local health departments making none. All but one of these HAT referrals were made to Nimule Hospital.

**Table 3:** Proportion of HCWs and facilities who made at least one HAT referral, before and after HAT training.

	HCWs		Facilities	
	N	%	n	%
**Ever referred before training (self-reported, baseline 1)**	30/113	26.5	19/56	33.9
**Referred in month before training (self-reported, baseline 2)**	14/113	12.4	11/56	19.6
**Referred in 4 months after training (surveillance)**	36*/97	37.1	35/49	71.4

*An additional 5 HCWs from 4 facilities made HAT referrals after being trained by HCWs who attended the workshop. If only HCWs who were followed-up are considered, the proportion who ever referred for HAT before the training was 25/97 (26.8%) and the proportion who referred in the month before training was 11/97 (11.3%). For facilities, these proportions were 16/49 (32.6%) and 9/49 (18.4%), respectively.

### Impact of intervention: Referrals made and received

#### HAT referrals from peripheral health facilities

Post-intervention HAT referral information was collected for 97/123 (78.9%) HCWs from 49/61 facilities represented at the training (Table 2). Evaluation visits were not completed with participating facilities because the trained HCW had left or could not be located (3 facilities), the facility had closed (4 private facilities) or time and accessibility constraints (5 private facilities). Because of time and accessibility constraints, no traditional practitioners and only 1/3 trained local authorities were visited.

In the four months after HAT training, 36 HCWs (37.1%, including 23 first-time referrers) and 35 (71.4%) facilities reported to have made at least 1 HAT referral, corresponding to a 1.4-fold and 2.1-fold increase in the proportion of referring HCWs and facilities, respectively, compared to baseline measure 1 (ever before referred for HAT, Table 3).

Collectively, HCWs reported referring at least 71 new patients for HAT. Two-thirds (48 or 67.6%) of referrals made after training were from HCWs who had never referred for HAT before. The increase occurred mainly in the predominantly Acholi payams most distant from the hospital (40 referrals or 56.3% made here). Whereas HCWs from non-public facilities were still more likely to make HAT referrals after training, public facilities contributed a greater number of referrals overall (50 or 70.4%). No public or military facilities reported receiving referrals from traditional practitioners who had attended the training (although it is possible that referred patients did not mention this).

Alternatively, if baseline measure 2 is considered (the volume of self-reported referrals made in the month before training), the mean number of monthly referrals after the training did not appear to appreciably differ from before the training (16 referrals (new or old, from facilities followed-up) reported for the month before training vs. 17.8 mean monthly new referrals after).

#### Referrals received at the hospital

**Figure 2:**
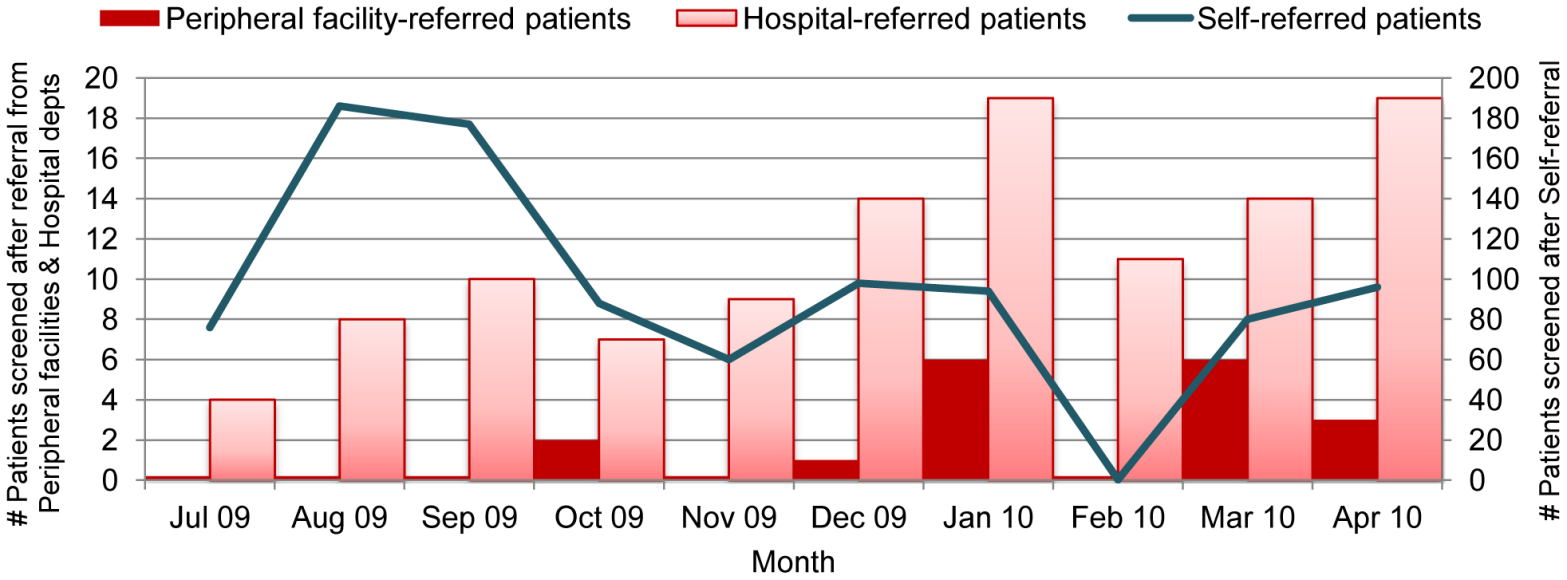
Source of referral for patients screened at hospital for HAT, by month of screening.

Before the intervention, less than 1% (2/527 or 0.4%) of people screened in the programme were referred from a peripheral facility (Table 4). After the training intervention, 13 people had been screened this way (13/272 or 3.7%), equivalent to a 6.5-fold increase in the rate of patients screened after referral from a peripheral HCW. This increase from a low base was observed despite an overall reduction in screening output at Nimule hospital (during a 7-week period between January and March 2010 the laboratory was only semi-functional, Figure 2). However, this large relative increase in screening of peripherally-referred patients is modest in absolute terms. The majority of patients screened after training continued to come through other referral routes, as did the majority of cases (2/24 cases detected after the intervention were peripherally-referred vs. 0/28 before). Notably, however, after training, 61.5% of peripheral HCW-referred patients came from areas outside of the hospital payam, whereas >90% of all referrals (mainly self-referred) continued to come from Nimule payam itself.

**Table 4:** HAT patient screening rates before and after HAT training, by referral route.

	Before training (Jul–Oct 2009)			After training (Dec 2009–Mar 2010)			After/before training rate ratio
Referral route	n	%	Rate/10,000 PY	n	%	Rate/10,000 PY	
**Self/community members**	527	93.8	91.3	272	77.3	47.1	0.5
**Hospital staff**	31	5.5	5.4	59	16.8	10.2	1.9
**Peripheral health facilities**	2	0.4	0.3	13	3.7	2.3	6.5
**Other/unknown**	2	0.4	0.3	8	2.3	1.4	4.0
**Total patients screened**	562	100.0	97.4	352	100.0	61.0	0.6

PY: Person-years of observation.

### Interpreting HCW referral behavior change

#### Changes in HCW HAT knowledge scores

Out of 123 participants who attended the training, 113 completed the pre-test, 110 the post-training test, and 55 the post-intervention evaluation test. Immediately after the training, there was a statistically significant increase in syndromic knowledge (participants associated a mean of 7.5/14 symptoms correctly with HAT in the pre-test and 12.6/14 in the post-training test). Despite a decrease in test scores between the training and evaluation (11.0/14), improvements in HAT knowledge from before the training remained statistically significant by the evaluation period 4 months later (Table 5).

**Table 5:** Mean overall test scores, before and after HAT training.

	n*	Mean symptoms correct	Mean test score out of 1.0 (SD)	Wilcoxon signed rank test p-value
Pre-training test	97	7/14	0.533 (0.265)	Pre vs eval <0.001
Post-training test	97	13/14	0.897 (0.112)	Pre vs post <0.001
Post-intervention evaluation test	52	11/14	0.787 (0.142)	Post vs eval <0.001

*In the pre-training test, 10/123 participants did not complete the test, 97/113 test-takers answered the question for all 14 symptoms. In the post-training test, 13/123 did not complete the test, 97/110 answered all 14 symptoms. In the post-intervention evaluation test, 68/123 did not complete the test, 52/55 answered all 14 symptoms. SD = Standard deviation.

Before the training intervention, several participant characteristics were associated with higher mean test scores (Table 6): working at a facility closer to Nimule hospital (p = 0.029), belonging to a non-Acholi tribe (p = 0.020) (Acholi indigenous land is farthest from the hospital), having a high patient workload (≥16 patients/day) (p = 0.024) and a higher level of formal clinical education (p = 0.072), as well as two measures of previous experience with HAT: ever having seen someone with HAT (p<0.001) and ever having referred a patient for HAT (p = 0.008). Encouragingly, however, training appeared to undo prior differences in HAT knowledge, with all but one of these associations (patient workload) losing significance at tests after the intervention.

**Table 6:** Associations between demographic variables and mean test scores.

	Pre-training test			Post-training test			Post-intervention evaluation test		
Variable*	n	Mean	p-value†	n	Mean	p-value	n	Mean	p-value
Gender									
Female	19	0.530	0.816	19	0.914	0.582	6	0.833	-
Male	78	0.534		78	0.893		46	0.781	
HCW age									
0–29 years	44	0.562	0.359	43	0.897	0.604	28	0.804	0.446
≥30 years	51	0.510		50	0.896		22	0.766	
HCW tribe									
Madi	31	0.601	**0.020**	30	0.888	0.288	18	0.833	-
Acholi	53	0.461		52	0.918		27	0.767	
Other	12	0.667		12	0.821		6	0.738	
HCW level of clinical training									
C: No formal	47	0.511	0.072	43	0.900	0.563	19	0.823	-
B: CHW	38	0.509		41	0.889		29	0.761	
A: CO/nurse	12	0.696		13	0.912		4	0.804	
Facility type HCW works in									
Public	68	0.560	0.155	66	0.908	0.194	47	0.783	-
All other types	29	0.470		31	0.873		5	0.829	
Distance of facility from hospital									
Hospital & neighbouring payams	38	0.615	**0.029**	37	0.871	0.1000	19	0.842	0.054
Distant payams	59	0.481		60	0.913		33	0.755	
Number of patients HCW sees/day									
0–15 pts	38	0.442	**0.024**	37	0.869	0.168	15	0.714	**0.004**
≥16 pts	58	0.586		55	0.910		35	0.814	
HCW had ever seen someone with HAT									
No	29	0.374	**<0.001**	30	0.888	0.634	16	0.759	0.440
Yes	68	0.601		65	0.899		35	0.800	
HCW had ever referred someone for HAT									
No	73	0.493	**0.008**	71	0.898	0.446	38	0.776	0.555
Yes	24	0.655		24	0.887		13	0.819	

*For differences by demographic variables in test scores at one time point significant at the 0.05 level, p-values are shown in bold font. Where p-values are not shown (-), numbers were too small for analysis of significance.

† Kruskal-Wallis tests were used to compare mean scores between demographic sub-groups.

**Figure 3:**
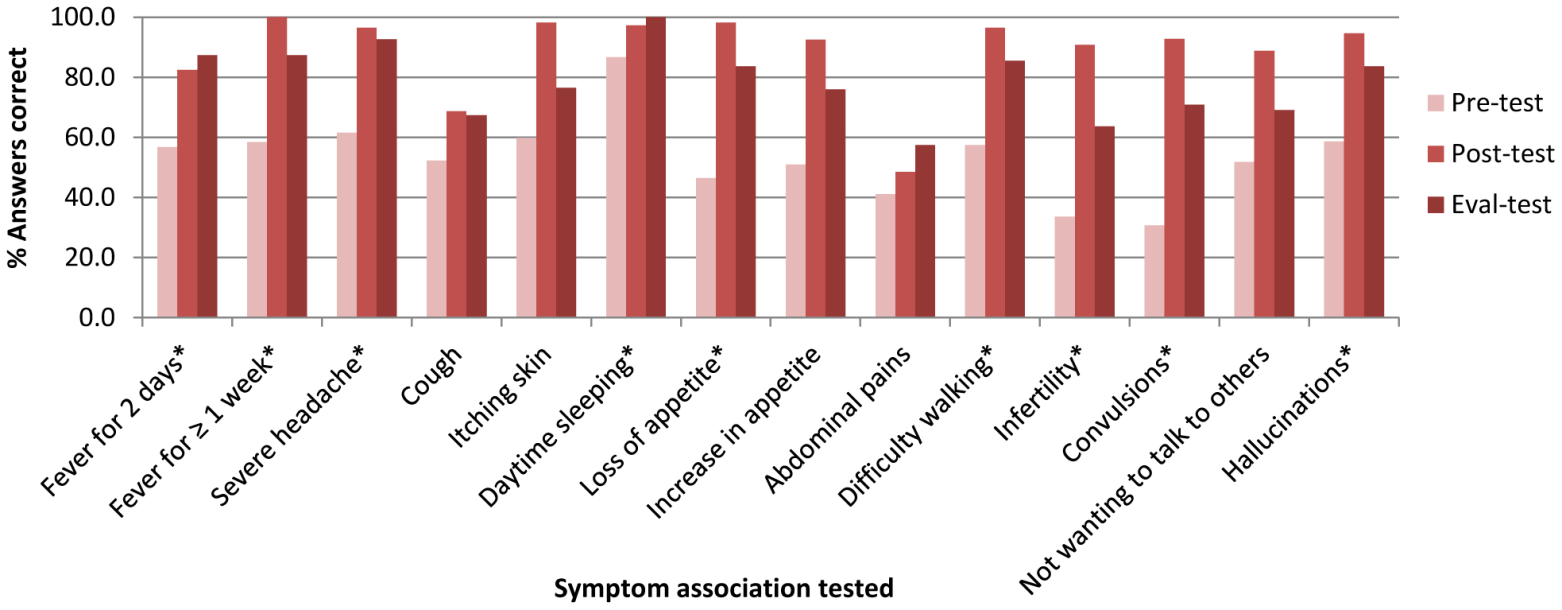
Distribution of correct test responses for individual symptoms, before and after HAT training. The numbers of people providing an answer for each symptom-association varied between tests. Pre-training (Pre-) test n = 107–113, post-training (Post-) test n = 106–110, post-intervention evaluation (Eval-) test n = 54–55. “Don't know” responses were categorised as incorrect. Fever for 2 days, cough and abdominal pains are not associated with HAT. *McNemar's p-value indicates a significant (p<0.05) increase in correct associations between pre-training and post-intervention evaluation tests.

Increases in correct test answers were observed for all symptom-associations tested in the evaluation period; increases for nine symptom-associations were statistically significant (Figure 3). The symptoms most associated with HAT (by at least 80% of participants) by the evaluation period were: daytime sleeping, severe headache, fever ≥1 week, difficulty walking, hallucinations (“seeing or hearing things that are not there”) and loss of appetite. Apart from appetite loss, these symptoms were also among the most associated with HAT before the training, indicating consolidated learning of pre-existing knowledge. Evidence of acquisition of new knowledge comes from significant improvement in knowledge scores for convulsions (which scored poorest in the pre-test) and two symptoms discussed in workshops in relation to problematic existing referral practices (short-term fever and loss of appetite). By evaluation, participants appeared least confident in ruling-out HAT when presented with symptoms not associated with the disease, as abdominal pains and cough (along with infertility which is associated with HAT) yielded the most incorrect answers by the evaluation period and increases were not significant.

#### Appropriateness of referrals

Of the 69 patients referred by peripheral HCWs for whom symptom data was available, all would have been referred by at least one of the expert clinician reviewers (only 1 patient would have been referred by less than 3 experts), indicating that most referrals made after the intervention may have been considered appropriate in syndromic terms. Fifty-seven (83%) of the patients referred by HCWs following training would also have been referred according to the focus-specific syndromic algorithm.

#### Circumstances of referral

During evaluation interviews, the most common route through which HCWs reported identifying patients for referral was through routine outpatient practice (34/65 or 52.3% of new referrals made, for which this data could be collected); however, identifying patients through informal, day-to-day interactions in the community was also common (14 or 21.5%), as was identification of patients at community meetings held by HCWs to discuss the symptoms of HAT (16 or 24.6%). HCWs were not specifically requested to conduct these types of meetings, but some reported initiating them spontaneously when their ability to see patients through formal facility-based consultations was limited, either because facilities did not yet exist in areas they were responsible for or because of a lack of drug supply.

In military barracks, formal procedures to investigate sickness (e.g. reasons for absence at morning parades) sometimes appeared to play a role in alerting HCWs to abnormal patient behavior, however patients were normally only considered for referral after long-term (≥1 month) informal observation (8 patients were identified by HCWs working in military barracks this way, 2 were identified after meetings held in barracks, 2 were identified at nearby private clinics).

#### Reasons for referral

Information on the reasons HCWs used to refer 54 new patients for HAT could be collected in the evaluation period.

In referrals, HCWs appeared to use symptoms and case detection/referral narratives conceptualised as both pre-existing and introduced by the training intervention, with pre-existing ideas predominating (Table 7). Among referral ideas that were pre-existing, HCWs cited abnormal sleeping behaviour (excessive sleeping or insomnia) as a consideration in two-thirds (36 patients or 66.7%) of referrals; abnormal mental behaviour ranging from a patient ignoring her baby to wandering in the bush at night was also considered in almost half of referrals (25 or 46.3%). Patient-reported changes to appetite and weight were also common (16 or 29.6% of referrals), but referrals citing appetite and weight loss were approximately as common as those citing an increase. Enlarged cervical lymph nodes were identified in a fifth of referrals (11 or 20.4%).

**Table 7:** Numbers of patients in whom new and pre-existing ideas about HAT case detection led to referral after training (n = 54).

Syndromic HAT case detection idea discussed*	Pre-existing idea reinforced by training n (%)	New idea introduced in training n (%)	Origin of idea unclear n (%)
Abnormal sleeping behaviour (daytime sleeping and/or insomnia) could be due to HAT	36 (66.7%)		
Mental confusion/abnormal mental behaviour (confusion, forgetfulness, aggression, hallucinations) could be due to HAT	25 (46.3%)		
Excessive appetite could be due to HAT	8 (14.8%)		
Weight gain could be due to HAT	2 (3.7%)		
Reduced appetite could be due to HAT		7 (13.0%)	
Weight loss could be due to HAT		3 (5.6%)	
Prolonged headache could be due to HAT	26 (48.1%)		
Prolonged fever (reported) could be due to HAT	16 (29.6%)		
Pains in the body could be due to HAT	11 (20.4%)		
Enlarged cervical lymph nodes could be due to HAT	11 (20.4%)		
Convulsions could be due to HAT		6 (11.1%)	
Neurological problems (difficulty walking/numbness is legs) could be due to HAT		4 (7.4%)	
Neurological signs (painful tibia) could be due to HAT		3 (5.6%)	
Fertility problem (miscarriage, pregnancy concern, lack of menstruation) could be due to HAT		3 (5.6%)	
Weakness could be due to HAT			8 (14.8%)
Very poor overall state of health could be due to HAT			5 (9.3%)
Other symptoms (night sweats, shaking, rash, coma) could be due to HAT			3 (5.6%)
Malaria- and typhoid-like symptoms unresolved by local diagnosis/treatment could be due to HAT	37 (68.5%)		
HAT can affect people who drink (or appear to drink) excessively		6 (11.1%)	
HAT can affect people who are imprisoned for strange/violent behaviour		3 (5.6%)	

*More than one referral rationale could apply to an individual patient.

Among HAT referral ideas that we believe were mainly new to participants, convulsions was cited in decision-making for just over 10% of referrals (6 or 11.1%). Evidence of neurological involvement (difficulty walking associated with leg numbness and tibia sensitivity –Kerendel's sign) was an important consideration in a small but noteworthy number (7) of referrals. Despite substantial discussion in workshops about the fertility complications possible in HAT, only three patients were referred because of this symptom.

Frequently identified in preliminary research, the most commonly reported narrative that led to HAT referral after the intervention was because HCWs had ‘ruled out’ malaria and/or typhoid locally (37 or 68.5% of patients). Setting aside problems associated with test specificity [25], [42], [43], as far as HCWs were concerned, they had effectively reported ruling out both diseases for 18 (33.3%) patients, either because patients had taken both types of treatments presumptively (10), patients had tested negative for both types of disease (2), or patients had taken treatment presumptively for one disease and tested negative for the other (6). A further two patients with severe disease were referred to Nimule hospital for urgent malaria, typhoid and HAT testing together. HCWs appeared to have routinely investigated previous malaria and/or typhoid history in patients presenting with severe symptoms (e.g. all 8 patients presenting with convulsions or hallucinations and all 5 patients with a poor overall state of health), but referrals after local malaria/typhoid diagnostic failure also appeared common in patients with more mild symptoms (e.g. patients presenting with prolonged back pains only).

Often these referral narratives were accompanied by statements that suggested HCWs otherwise felt at a loss to assign a diagnosis, such as “I just give her analgesics every week [for severe headache]” (*patient 71, public facility referral*) or “I have given her treatment for malaria and typhoid many times but it only relieves her symptoms for about four days, then she starts complaining again” (*patient 78, public facility referral*), suggesting that local diagnostic failure was the main reason to consider HAT. This rationale was reportedly also explained to patients: “I told her, you have already tried malaria and typhoid treatment but it has all failed, so now you need to go to Nimule” (*patient 66 with mental confusion and convulsions, public facility referral*). At times, HAT-like symptoms such as enlarged lymph nodes (*patient 24, public facility referral*) or abnormally aggressive behaviour (*patients 7 & 8, public facility referrals*) appeared to have been recognised only once HAT referral was considered because of local diagnostic failure, supporting HCWs' suspicions. At others, HAT-like symptoms themselves seemed to be the driving reason behind referral as for one patient who a HCW observed sleeping in his waiting room, prompting consideration of HAT (*patient 4, public facility referral*). This patient was told to self-treat for malaria at a private clinic and then go to Nimule if his symptoms persisted.

Interestingly, HCWs appeared less likely to report considering local malaria/typhoid failure in referral decisions when patients were identified outside formal consultations. Whereas malaria/typhoid failure was considered in the majority (26/30 or 86.7%) of referrals made at facilities and in two-thirds (6/9 or 66.7%) of referrals after community meetings, it was considered in only 28.6% (4/14) referrals initiated by HCWs' informal observations of people they knew in their communities. HAT referral after local diagnostic failure was common no matter the distance from Nimule Hospital.

The two narratives introduced in the training associating HAT symptoms with drunkenness and imprisonment appeared to be of value in referring male, especially soldier patients; HCWs made seven referrals based on these. Rationale which supported referral decisions tended to incorporate assessments of what constituted atypical (i.e., caused by HAT) drunk behaviour such as, “When he drinks he always wants to fight, even if he drinks only a little” (*patient 56, also patient 41, military barracks referrals*), “once he takes a little drink like this then he's straight to sleep” (*patient 40, military barracks referral*) and even, “he moved like he was drunk, even though he does not drink” (*patient 8, private clinic referral*).

HCWs in both interviews and group discussions directly attributed referrals made to the training intervention with supportive statements such as, “Before, everybody thought it was just drinking,” (*patients 41 & 56, military barracks referrals*) and, “Before this training, we were treating all these people for just malaria. Now, with these community meetings, we see all these symptoms of sleeping sickness in our people and we think, actually, there is a lot of sleeping sickness here” (*public facility HCW from Magwi FGD*).

### Interpreting referral completion

#### Referrals completed

**Figure 4:**
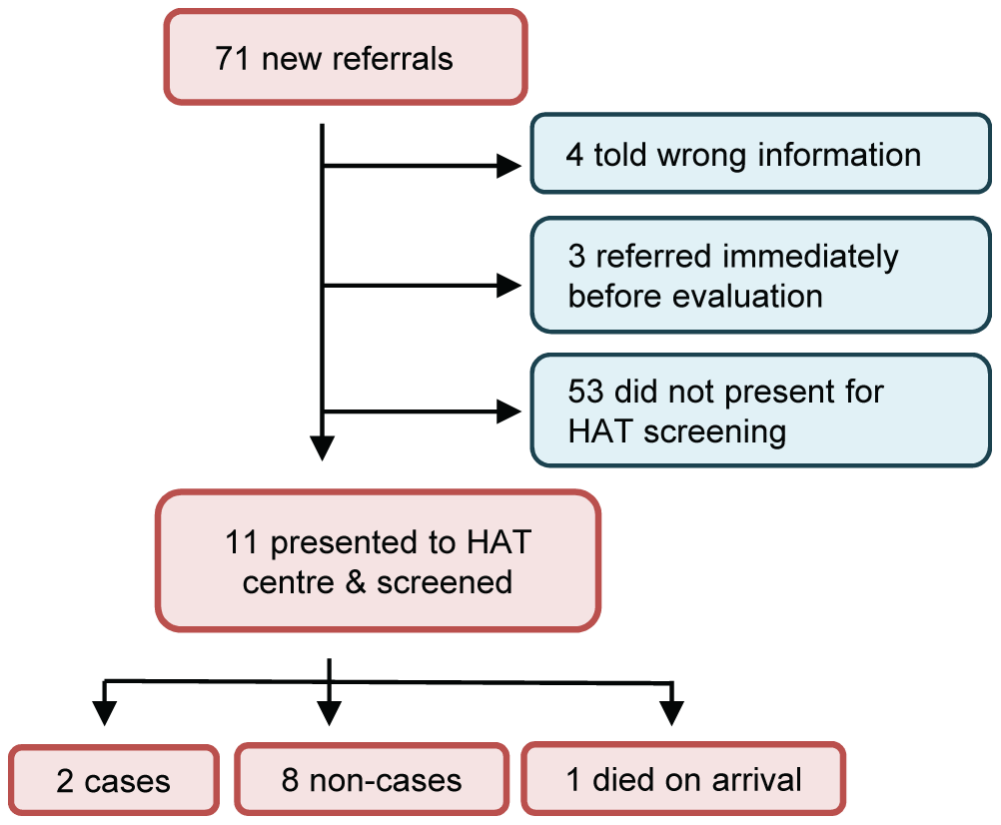
Screening outcome of new referrals made.

Of the 71 new referrals made after the training, only 11 (15.5%) referrals were received at the hospital and screened (Figure 4). Referrals were received at the hospital a median of 3 weeks after referrals were made (range 1–25 weeks). Of those patients who completed their referral, 2/11 (18.2%) were confirmed to be cases and were treated for HAT; a third patient died in the hospital before a HAT test could be completed.

Referral completion was significantly associated with higher levels of clinical education in referring HCWs (40% completion of referrals made by COs and nurses vs. 10.2% by other cadres) and with non-public facilities (31.6% completion vs. 11.1% in public facilities). Although numbers were small, shorter distances to Nimule hospital appeared to be associated with referral completion. In payams most distant from the screening centre, only 5% (2/36) of patients completed referrals while in payams less than one day's walk away, one-third (9/28) of patients completed referrals during the follow-up period (risk ratio 5.8, p = 0.007).

#### HCW accounts of referral non-completion

Of the 60 referrals who had not presented to the hospital by evaluation visits, 4 patients were mistakenly told by referring HCWs to wait until HAT AS teams came to the village. Common reported reasons for non-completion of referrals were explored with HCWs through discussion of an additional 32 referrals.

Long distances from Nimule hospital and the lack of affordable local transportation opportunities were mentioned often and discussed at length by HCWs in interviews and discussions as a critical barrier to patient referral completion (cited as a likely problem in 20 or 62.5% of uncompleted referrals, Table 8). Both private and public transportation in Magwi County is expensive; for patients outside of the hospital payam, a return journey for one person by private means cost between USD 8-84 (depending on payam distance, using the most common method) in 2010. There was only one government ambulance and few NGO vehicles available to transport patients to the hospital. In group discussions, HCWs believed only a small minority of patients disagreed with the HAT referral or sought treatment from a non-HAT centre. Instead, frequent HCW interview statements such as, “he is waiting to go” implied that HCWs recognised patient willingness to complete the HAT referral and implied a lack of blame for patients not *yet* being able to, because of these insurmountable structural barriers.

**Table 8:** HCW-reported reasons why referred patients did not present for HAT testing (n = 32).

**20 patients (62.5%): Cost or scarcity of transportation**	
18	Patient complained of lack of transportation or associated cost
1	Family member was waiting to be paid before paying for patient transport
3	Patient treated unsuccessfully after travelling to a hospital before HAT referral & patient therefore reluctant to go again
**7 patients (21.9%): Non-medical travel**	
7	Patient left county for a reason unrelated to illness, including 2 barracks transfers
**5 patients (15.6%): Insufficient family support**	
3	Patient has psychiatric problems and has no carer available to travel with
2	Household responsibilities make it difficult for patient to travel
**4 patients (12.5%): Permission to travel denied**	
4	Military refused permission to travel
**4 patients (12.5%): Non-HAT diagnosis treatment pursued**	
3	Patient went to non-HAT referral hospital
1	Family disagreed with HAT diagnosis & preferred to treat with herbs
**3 patients (9.4%): Hospital patient processing problem implicated**	
3	Patient reported to have travelled to Nimule since referral, but no record of hospital visit

Note: Multiple reasons may be listed for individual patients.

‘Poverty’, at times related to the hardships of the return migration process, was a related concept frequently cited by HCWs in the most distant areas, and was almost always directly linked to the difficulty of obtaining cash for independent transportation. Even with their own family members, HCWs found it difficult to convince patients to complete referrals because of the cost of transport: “My sister-in-law, I went to counsel her in the compound but she just came back to me, ‘Where is the transport? Give me the transport!’ So it is difficult for us here because we are only volunteers [referring to the fact that most HCWs go unpaid by the Ministry of Health for several months at a time]” (*patient 78, public facility referral*).

Patients were reportedly waiting for any of several events that might support referral completion: for opportunities whereby patients could obtain cash to pay for private transportation, for referring HCWs to help them access free transportation in government ambulances, military or NGO vehicles, or for the acuity of symptoms to become so serious that access to paid or free transport would be necessitated. Of the 11 patients who successfully completed referrals during the intervention period, two patients' transportation was organised by the military, a HCW opportunistically advocated for one patient to be allowed in the government ambulance when it was transporting someone from a road accident, another HCW radioed to a local NGO to pick-up one patient and the remaining (7) patients used personal funds to complete the referral.

In theory, medical NGOs in South Sudan are willing to transport patients when they have space in vehicles during operational movements. However, in group discussions, HCWs revealed that in practice, only HCWs at facilities directly supported by these NGOs tend to liaise with NGO drivers to link patients with transportation, leaving the majority of HCWs at government-only-supported (or private or military) facilities with no regular access to these opportunities. This lack of an organised, accessible patient transportation system in the county therefore largely puts the onus of referral completion on the patient. As one referring HCW saw it, “they [patients] are the one to decide” (*HCW2, private facility, Pageri FGD*) whether referrals are completed, but most patients lack the autonomy to be able to act on intentions to complete them.

While distance and travel costs were considered important barriers in the most distant payams, HCWs reported to have discussed this problem with only 2/20 patients referred from payams neighbouring Nimule and none from Nimule, itself. In areas closer to Nimule, opportunity costs therefore potentially took on increasing importance, such as the need to choose between travelling to the hospital and meeting family responsibilities such as child-care and agricultural work by both patients and their carers (especially for patients with psychiatric symptoms) (5 patients). For three patients who had apparently managed to travel to Nimule hospital for treatment-seeking purposes, no record of any hospital visit since referral was available, potentially implicating patient reception and processing problems in the hospital, possibly during the months when HAT lab services were restricted.

In military populations, 12 new soldier patients were referred by HCWs in barracks medical corps or in facilities near barracks. Only two patients, both recognised by medical corps workers, appeared to receive military support for transportation to complete referrals; both were described as being taken “by force” because they were mentally-affected. In the other cases, permission to travel even by other means, or permission to delay transfer to another barracks farther from HAT treatment centres was not unilaterally given. To explain decision-making about patient travel in the military, two ‘laws’ relevant to soldier patient welfare were discussed in interviews that were occasionally in contradiction. The first referred to the primary purpose of soldiers being in barracks, which was to work or receive training: “According to [the] law, someone who is training, they cannot be given a departure order, a permit to leave” (*patients 9 & 10, military barracks referrals*). At the same time, as a military commander from this barracks explained the second law, “If soldiers are sick, we cannot use their bodies for work and it will be on them [commanders] if they do not help these people.” In practice, it was non-medical military authorities who appeared to balance these laws, making judgements about how sick a patient was when deciding whether or not to release soldiers from duty and grant travel permission. Referral letters from medical personnel were taken into consideration but, like civilian patients who ‘wait to go’, commanders were also discussed as ‘taking time’ to grant departure orders to weigh a patient's symptom severity against their other responsibilities to the military as a whole.

A final potential barrier to referral completion identified only in group discussions related to HAT-associated stigma, especially in the distant payams of the county which had comparatively less exposure to HAT screening activities. Owing to the fact that HAT was not known to exist in this area before the war, HCWs also occasionally referred to HAT as a “new disease” during the training and evaluation interviews. This was similar to the way in which HIV/AIDS was commonly referred to, another disease initially identified in this population during war-time (*Magwi, Lobone and Nimule FGDs*). HAT may therefore have suffered from some AIDS-associated stigma there and we notably corrected two HCWs here during evaluation interviews who mistakenly believed HAT to be sexually transmitted. HCWs suspected that patients did not yet know enough about HAT to recognise symptoms, or about the transmission and treatability of the disease to subsequently seek specific advice about HAT from a HCW (*Magwi & Lobone FGDs*). HCWs suggested this may have been a reason why some potential cases did not self-identify after community HAT meetings, but HCWs were unsure whether this would prevent someone from completing a referral.

## Discussion

This study evaluated the effect that a simple intervention, training of peripheral HCWs to recognise and refer potential syndromic cases of HAT, could have on improving PS rates in rural patient populations in MC. We evaluated this intervention in several ways which suggest that it had a positive effect on HCW referral practices, but a quantitatively marginal effect on passive case-detection largely because of failure to complete referrals by patients.

Against a low baseline of HCW HAT referral experience (only a quarter of HCWs trained in a third of facilities had ever made a HAT referral before), the intervention appeared to have increased the numbers of peripheral HCWs and their distribution in facilities across the county who might now regularly consider HAT in their differential diagnoses of patients. HCWs in almost three-quarters of facilities surveyed in the county had made at least one HAT referral in the four months since training. Two-thirds of the 71 referrals made after the intervention came from new referrers. This increase was especially notable in HCWs working in areas most distant from the screening facility who probably had less exposure to the HAT programme's active and passive screening activities.

In tests, HCW knowledge of the signs and symptoms associated with HAT disease was significantly improved across all demographic sub-groups of participants such that, after the intervention, any differences in HAT symptom knowledge before the training intervention became non-significant. This improvement in knowledge suggested that there was need for HAT training on symptom recognition in all groups of HCWs in the county and that the HAT training package was delivered at a level that was accessible to all participants, regardless of their level of formal clinical training and experience with the disease. Knowledge scores for HAT symptom associations conceptualised in preliminary research as ‘pre-existing’ in HCW HAT case detection narratives were generally higher than for associations conceptualised as ‘new’ or introduced by the training but improvements in both types of knowledge indicated both consolidated learning and expanded knowledge across a range of HAT symptoms.

When syndromic knowledge was translated into referral practice, the referrals made after the intervention appeared to be syndromically appropriate, based on expert review. Qualitative interviews suggested that in these, HCWs used both pre-existing and new ideas about how to identify likely cases of HAT syndromically. Numerically, pre-existing ideas tended to predominate. Considering the relative frequency with which particular signs and symptoms might be expected to occur in a typical, mainly adult patient population, less use of some of the ‘new’ ideas about HAT introduced in workshops might naturally be expected (e.g., convulsions and neurological problems vs. mental confusion and headaches). Fertility problems, however, were probably an example of a new concept that was under-utilised by HCWs in referrals; furthermore, almost 2/5 HCWs tested in the evaluation period did not correctly associate this symptom with HAT, suggesting need for further exploration and discussion of the significance of HAT-fertility concepts with HCWs.

When HCWs were approached by patients at facilities or after community meetings, differentially ruling out malaria/typhoid was a very common but complex HAT referral behaviour. Often, HCWs' desire to resolve failed malaria/typhoid diagnoses appeared to be the driving reason behind HAT referral, prompting HCWs to specifically look for HAT-supportive signs such as enlarged lymph nodes. In severe cases, malaria/typhoid was always ruled out but we could not determine in this study whether this potentially acted as a barrier to earlier consideration of HAT [20], [24]; preliminary research by us suggested that peripheral HCWs considered this highly appropriate. Outside of formal consultations, HCWs less commonly relied on malaria/typhoid differential diagnoses to make HAT referral decisions, suggesting that HCWs may gain confidence in making syndromic referrals with an extended period of observation.

Along with expanding and consolidating existing syndromic HAT detection knowledge, we believe that the training intervention also empowered HCWs to think of HAT as more common in MC and as an appropriate syndromic diagnosis to make at the peripheral level, supporting translation of knowledge into practice (Figure 1). Evidence for this comes mainly from the increased numbers of HCWs who made a referral but who never had before, coupled with supportive statements by HCWs about what they learned from the training. Many HCWs proactively adapted their working practices to compensate for reduced patient attendance, for instance, when stock-outs of medicines were frequent or prolonged, such that identification of patients occurred outside of formal healthcare facilities as often as it did inside of them. HAT referral also appeared to be particularly accommodated as a useful differential syndromic diagnosis that could be referred on when malaria or typhoid was ruled out, as already discussed.

The training intervention largely, however, failed to empower patients to complete HAT referrals made in the periphery (or HCWs to meaningfully support the process), under the existing referral system's constraints. Despite a 6.5-fold relative increase in this type of referral received at Nimule Hospital attributable to the HCW training intervention, in terms of the absolute numbers of patients screened, this increase was modest. A third of patients referred for HAT from areas close to the hospital eventually presented for screening, but in areas more than one day's walk away (half of the county), only a very small minority of patients who were referred in the intervention presented.

Long distances and the lack of affordable transportation opportunities for patients were probably the most important barriers preventing patients referred for HAT from reaching the hospital for screening, as in other studies of centralised health service use in South Sudan and rural African settings [44]–[50]. Relatedly, the fact that patients referred from private and military facilities were more likely to complete referrals suggested that relative wealth may have been important, since private facility services (tests, drugs) cost money and soldiers receive government salaries. Treatment costs and other factors have been used to explain decisions by households who ‘wait to go’ in malaria studies [31] and transportation subsidies with community-based counselling have successfully increased referral completion in HIV programmes in Africa [51]. Although not measured in this study, higher household wealth and maternal education (of patients or primary care-givers) have been associated with increased maternal and child health care-seeking in South Sudan [52] and may have been important in patient referral completion here. Referring HCWs were rarely able to successfully support the referrals process (for instance, by assisting patients to secure transportation or advocating for soldiers to obtain departure orders) but quantitative evidence suggested that individual HCWs' level of clinical education may have influenced this process, as elsewhere [49], [53]. Specific ideas introduced in the training about considering HAT as a diagnosis for patients who commonly appear drunk or are imprisoned for strange/violent behaviour were used by HCWs who commonly interact with military populations. Given the apparent difficulty that military HCWs faced in advocating patient referrals, however, further intervention might be needed to support HCWs in discussions with commanders to resolve some of the uncertainty that leads to potential disagreement in this context [54]. Further study of referral completion from the perspective of patients might also generate other critical barriers and opportunities to support completion not identified here.

The planned introduction of HAT RDTs [14], improved microscopy techniques [55] and potentially safe oral medicines [56] appropriate for use in primary and secondary facilities offer a promising new approach to bring screening, diagnosis and treatment services closer to patients and HCWs in peripheral areas like MC in South Sudan. Offering syndromic training or implementing syndromic algorithms may help guide the rational use of RDTs as the first step in case detection algorithms and this study suggests that educational training can be used to augment peripheral HCW HAT syndromic referral practice. The implementation of either peripheral HCW-controlled syndromic or RDT technologies might conceivably also synergistically empower HCWs to make more syndromic referrals in the first place. In an elimination scenario, involving health workers in the periphery, close to the last patients, will also be essential to monitor the absence of new syndromic cases. As this study suggests, substantial infrastructural challenges related to the ruling out of other common diagnoses and management of HAT RDT-positive patients who need access to hospital-based confirmatory tests will need to be addressed.

### Limitations

We could not follow-up all HCWs (or any traditional practitioners) to identify all peripheral referrals made. The opportunity for patients to complete their referrals was limited to within four months of the training, regardless of how long after training that referral occurred, and completion could be associated with season. Furthermore, we had to rely on referring HCWs' accounts to understand reasons behind those uncompleted referrals.

A greater overall effect on passive case detection might have been observed in a higher prevalence scenario, where more syndromic referrals might have been made, even if referral completion rates remained the same. This suggests an even greater potential benefit of intervention for more highly endemic areas such as the Democratic Republic of Congo. Under-recognition of HAT symptoms in practice likely continued to be a problem in this study but we could not directly assess syndromic case detection performance during clinical encounters without changing clinical practice. Determining missed opportunities for referral in this circumstance would have been difficult, as no ‘gold standard’ tool (such as a highly discriminating syndromic algorithm [36], [57]) exists against which to evaluate HCW syndromic recognition of patients in need of HAT screening.

In this environment where peripheral HAT referral was uncommon, identifying a specific new *type* of referral behaviour (HAT referral ever made, baseline measure 1) appeared more informative and valid than retrospective estimation of monthly referral rates, using HCW recall without reference to records or a key timeline of events (baseline measure 2). If we take as true that two-thirds of referrals made after the intervention were by new referrers, three explanations are possible to explain the difference in effect the intervention appeared to have on baseline HCW referral behaviour: the monthly rate of referrals made by experienced referrers substantially decreased after the intervention, the estimated pre-intervention rate consisted mainly of ‘old’ referrals, or the pre-intervention rate was substantially over-estimated on questionnaires. We believe the latter explanation to be most plausible. Despite these limitations, which are inevitable in this kind of evaluation in South Sudan, this study is the first to evaluate the effect of peripheral HCW training on HAT referrals to a PS service.

### Conclusions

In an era when approaches to HAT case detection and control must increasingly be integrated into health referral systems, it is important to understand the opportunities and challenges associated with syndromic case detection in first line facilities to design effective interventions. This is important now, and will also be important when RDTs for HAT become available in peripheral areas to assess whether they can be targeted to the right patients. In this training intervention in South Sudan, we were able to increase the numbers of peripheral HCWs who now identify potential cases and refer for HAT by expanding and consolidating pre-existing syndromic HAT detection knowledge and by encouraging consideration of HAT as a diagnosis that is appropriate to make at the peripheral level. Barriers to patients reaching facilities for confirmatory testing remain. Additional interventions will be required to improve peripheral HCW and patient access to screening, diagnosis and treatment to contribute meaningfully to HAT control under a passive case-detection approach. The advent of rapid diagnostic tests, if they prove useable in peripheral settings, may provide part of this solution.

## Supporting Information

File S1 **Syndromic HAT training HCW manual.**
(PDF)

File S2 **Syndromic HAT training HCW questionnaire.** Pre-intervention questionnaire completed by all HCWs who attended training, including HAT symptom knowledge association test.
(PDF)
