# Supplementary material for: A Mixed Methods Study of a Health Worker Training Intervention to Increase Syndromic Referral for Gambiense Human African Trypanosomiasis in South Sudan
Source: PLoS Negl Trop Dis. 2014 Mar 20;8(3):e2742. doi: 10.1371/journal.pntd.0002742 (PMC3961197; doi:10.1371/journal.pntd.0002742)
Supplement: File S1 — Syndromic HAT training HCW manual. (PDF) [file pntd.0002742.s001.pdf]

Merlin South Sudan
Health worker training manual:

Identification of signs and symptoms of Sleeping Sickness

Prepared by Elizeous Surur & Jennifer Palmer
October 2009, Nimule, South Sudan

Workshop schedule

Time	Facilitator	Activity
10:30 – 11:00		Participant registration, name tags, manual distribution
11:00 – 11:15	ES & JP	Welcome, introductions, workshop objectives
11:15 – 11:45	JP	Pre-workshop questionnaire
11:45 – 12:15	ES	Lecture I: Sleeping sickness transmission, distribution and control
12:15 – 1:00	ES	Lecture II: Signs & symptoms of sleeping sickness
1:00 – 1:30	ES	Lecture III: Testing and treatment
1:30 – 2:15		Lunch
2:15 – 2:45	JP	Presentation: Sleeping sickness & health workers in Magwi County
2:45 – 3:15	Volunteer	Tips on taking good patient histories
3:15 – 3:45	JP	Hands-on training: Completing referral logs
3:45 – 4:00	Volunteer	Tips on counselling for referral
4:00 – 5:30	ES	Patient role-plays & sleeping sickness symptoms memory games
5:30 – 6:00	JP & ES	Discussion about March 2010 Evaluation day workshop and informal Q & A
6:00 – 6:15	JP	Post-workshop questionnaire

1. Workshop objectives

The objective of this training programme is to train or re-train all of the health workers in Magwi County on how to recognize symptoms of the disease, sleeping sickness, and refer patients they think might have sleeping sickness to Nimule hospital for a test. We hope that by doing this, we will be able to increase the number of patients with sleeping sickness who are detected so that they can be treated and cured.

We will evaluate the success of the training workshop in 3 ways:

- using pre-workshop and post-workshop questionnaires to assess knowledge gained today, as well as knowledge retained 3-4 months after the workshop
- asking participants to keep logs of all patients that they refer for a sleeping sickness test in the next 3-4 months
- inviting participants to attend an evaluation day in March 2010 to submit their referral logs and participate in group discussions about their experiences and any difficulties they may have faced in identifying and referring patients

We may also talk to some of your patients on the evaluation day in March to understand their experiences with the referral process.

2. Introduction to sleeping sickness and its control

2.1 Causative organism

- Sleeping sickness is cause by a flagellated protozoan parasite called a trypanosome. Another name for sleeping sickness is human African trypanosomiasis (HAT).
- Dr David Bruce first identified the parasite in wild animals in Zululand in South Africa a century ago. After his discovery, another subspecies of parasite that was morphologically similar was identified in a European man in Gambia, West Africa. The two subspecies of sleeping sickness parasites were then called *Trypanosoma brucei (T.b.)*, named after Dr Bruce.

  The two types of sleeping sickness are:-

  A) T.b. rhodesiense
  - This is the east African type of sleeping sickness.
  - It is an acute form of sleeping sickness. It follows an acute progression, with clinical presentation of symptoms and signs emerging only weeks and months before death.
  - Patients with this form of disease may die in early stage (stage I) due to myocarditis (inflammation of the heart muscle).

  B) T.b. gambiense
  - This is the west African type of sleeping sickness and the type that is present in South Sudan. It is responsible for 90% of sleeping sickness infections in Africa.
  - It is a chronic form of sleeping sickness.
  - It has a chronic, progressive course of clinical presentation that takes months to more than two years to develop.

- Some patients in endemic areas are found to be asymptomatic (without symptoms) for years before they get sick.
- Untreated patients eventually die of infections, malnutrition or when the disease involves the central nervous system (CNS: brain and spinal fluid become infected with the parasite).

2.2 Transmission

a) The disease is mainly transmitted by an infected tsetse fly when it takes a blood meal

- Both male and female tsetse flies transmit the parasite.
- These tsetse fly vectors are in the genus *Glossina* and they are only found in Africa.
- Transmission of *T.b. rhodesiense* occurs in savannah grassland, and reservoirs of this disease are man, as well as wild and domestic animals.
- Transmission of *T.b. gambiense* occurs in small streams, near forests, lakes and rivers. As such, the vectors are sometimes called riverine tsetses. This vector needs high humidity and shade for its habitat. Some animals can act as a reservoir of this disease, but *T.b gambiense* infections are mostly found in humans.

b) Congenital transmission

- Transplacental transmission of sleeping sickness parasites occurs in pregnancy to the foetus.

c) Blood transmission

- Transmission during blood transfusions is also possible.

2.3 Epidemiology

- Sleeping sickness is distributed in a very large part of Sub-Saharan Africa: it is found in 36 African countries.
- It is an emerging public health crisis, 60 million people are at risk of infection.
- It is estimated that 50-70,000 new cases occur each year in Africa.

Figure: Regions where sleeping sickness is endemic in Africa

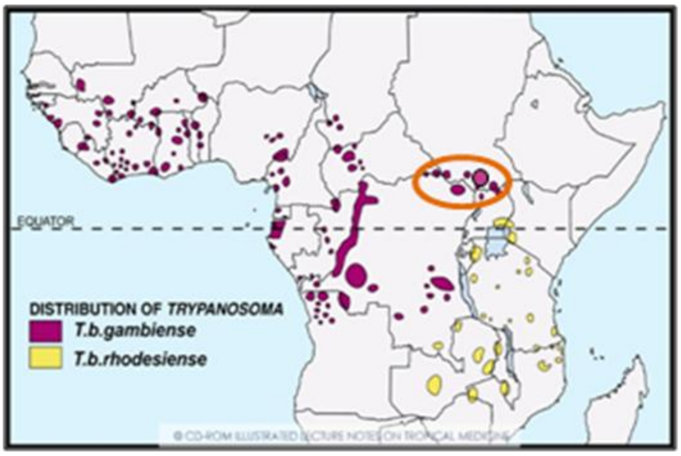

- *T.b. rhodesiense* is distributed from Ethiopia in the East to Botswana in South East Africa.

- *T.b. gambiense* extends from West to Central Africa including Southern Chad and South Sudan, DR Congo and down to Angola.
- In Uganda, *T.b gambiense* overlaps with *T.b rhodesiense* in the south eastern part of the country near lake Victoria.
- Highly endemic areas of sleeping sickness occur in remote rural areas which receive low priority in terms of health infrastructure resources by politicians and health officials. In addition, community-based care centres (like PHCUs) are considered the best way to address common public health problems like maternal and child health. Single disease-focused control activities are often considered too expensive. However, for sleeping sickness, this approach is considered the best way to control the disease. That is why we call sleeping sickness a "neglected disease".
- In the late 1990s there were epidemics of sleeping sickness in South Sudan, Uganda, DR Congo, Tanzania and Angola. Epidemics in these countries were associated with economic decline, civil disturbances, wars and refugee and population movements.

3. Signs & symptoms of sleeping sickness

3.1 Haemolymphatic stage (Stage I)

This is an early stage of sleeping sickness where the parasites are found in the blood and lymphatic system only.

This stage has **non-specific** clinical symptoms and signs which are:

- **Intermittent fever** lasting for about a week associated with waves of parasitaemia (increased numbers of parasites in the blood)
- **Headache**
- **Malaise** - generalised body weakness
- **Myalgia** –generalised muscle pain
- **Arthralgia** – generalised joint pain
- **Pruritis** – itching of the body
- **Anorexia** –loss of appetite
- **Increased appetite** – less common than loss of appetite
- **Asthenia** – weight loss due to muscle atrophy
- **Oedema** –swelling in the face and limbs
- Patients often develop mild normochromic anemia
- **Cervical lymphadenopathy**, especially at the posterior triangle of the neck (**Winterbottom's sign**). These nodes are rubbery, fixed and non tender. *This is the only stage I symptom that can be considered specific to *gambiense* sleeping sickness*
- **Splenomegaly** - in many cases spleen is enlarged to moderate size
- **Hepatomegaly** - enlargement of the liver
- Cardiovascular disorders, eg **heart palpations** or pericardial pain
- Renal manifestions, **Albuminuria** - in this case patients may sometimes mistakenly be treated for nephrotic syndrome

- **Intercurrent infections** - e.g lung infections (**pneumonia**). Sometimes patients die of these infections but in most cases, death follows in the late stage when the disease involves the CNS

3.2 Meningoencephalitic stage (Stage II)

This is the late stage of sleeping sickness disease when parasites have already invaded the cerebral spinal fluid (CSF) and the brain. Therefore, the clinical symptoms and signs of this stage present with:

I. SLEEP & CONSCIOUSNESS DISORDERS

- **Insomnia** - Nocturnal sleeplessness (cannot sleep at night)
- **Daytime somnolence** - Excessive day time sleeping
- **Agnosia** – Patient has reduced cognitive function (**confusion**) or forgets quickly (**amnesia**)

II. MOTOR & TONE DISORDERS

- **Tremor** – in the hands and lips (Castelloni's sign)
- **Ataxic gait** - patients appear to have Parkinson's disease with shuffling walk
- **Slurred speech** – speech impairment
- **Malaise** – general weakness such that patient is inactive or withdrawn from normal social activities
- **Dyskinesia** – disturbance of steadiness
- **Chorea** –abnormal movement of joints and face
- **Hypertonia /Hypotonia** –extreme extension of muscles or arteries
- **Myoclonus** - abnormal reflexes or muscle movements
- **Hemiplegia** – paralysis of one side of the body
- **Paraplegia** –paralysis of both sides of the body
- **Convulsions** – generalized or localized fits

III. PSYCHIATRIC DISORDERS

- **Aggression** and paranoid state
- **Schizophrenia** – patient may present with visual or auditory hallucinations (hearing or seeing things that are not there)
- **Romberg's sign** – patient does not stay in one place (wanders around)

IV. DISORDERS OF THE SENSES

- **Hyperesthesia** – e.g. Kerandel's sign, this sign is detected at the tibia prominent (shin bone) by pressing it firmly, patient then feels this very painfully. Alternatively, when a patient tries to open a padlock with this sign he/she feels severe pain

V. NEURO-ENDOCRINE DISORDERS

- **Impotence -** sexual dysfunction, male patient fails to achieve erection
- **Amenorrhoea** - female patient stops having regular menstruations
- **Abortion** - expulsion of unborn foetus from uterus
- **Infertility** – in both males and females

3.3 Disadvantages of not treating sleeping sickness

- 100% of untreated patients will die. There is no immunity against this disease. There is currently no vaccine against sleeping sickness.
- Sleeping sickness can break up families, when it causes psychiatric problems and infertility.
- When sleeping sickness affects large numbers of the population in villages, as in outbreaks, it affects the village's economy, because so many adults are too sick to work.

It is therefore important that healthcare workers who identify signs and symptoms of sleeping sickness in their patients spend some time counselling patients about the disadvantages of not treating this disease (and not treating it early) to ensure that the patient will present at the testing and treatment facility for a sleeping sickness test.

4. Principles of *T.b. gambiense* control

4.1 Case finding and treatment

- This is the most important element of sleeping sickness control because it saves patient lives and also controls the spread of disease in a community.
- There are two main ways to detect cases: passive screening (patient is detected at the hospital) and mobile / active screening (patient is detected by mobile teams in their village). Once identified, all patients are treated and followed-up after 6 months, 12 months and 24 months before they are declared cured.
- Active screening is very important to control sleeping sickness caused by *T.b gambiense*, especially in outbreaks.

4.2 Vector control to reduce human – fly contacts

- <u>Bush clearance</u>: Clearing bush and felling trees around water contact points and crossings can achieve this. Clear 20 m x 200 m stretches of land on each side of the river bank.
- <u>Use of tsetse traps or targets</u>: Blue and black-coloured cloth traps and targets impregnated with insecticide are usually placed at points of high human-fly contact over a wide-spread area.
- <u>Aerial spraying</u>: Spraying insecticides from airplanes can achieve a very rapid reduction in tsetse fly populations and is commonly used for *rhodesiense* sleeping sickness, but it is expensive.

5. Diagnosis of Sleeping Sickness

5.1 Card Agglutination Test for Trypanosomiasis (CATT)

- This is a diagnostic test for the detection of sleeping sickness caused by *T.b. gambiense* in blood. It is the first screening test used in either type of screening.

- The test detects the circulating antibodies which are produced following infection by the parasite. These antibodies are demonstrated in whole blood, serum or plasma by an agglutination test on a plastic card. If there is agglutination, it is a positive result.

5.2 Cervical lymph node puncture and aspiration for microscopy

- Infection of *T.b. gambiense* leads to enlargement of the cervical lymph nodes especially at the posterior triangle area of the neck (Winterbottom's sign).
- Infected nodes are rubbery, fixed and painless.
- Collection of lymph node fluid from these nodes for analysis requires using aseptic technique.
- Patients are asked to sit comfortably before lymph node fluid is aspirated using a sterile syringe and the fluid is put on a slide with a coverslip, then examined under a microscope.
- A positive result shows rapid movements of trypanosomes with the help of their undulating membrane and flagella between white blood cells.
- This is parasitological confirmation of infection with *T.b. gambiense* .

5.3 Microhaematocrit capillary tube centrifugation technique (WOO Test)

- This is a procedure for parasitological diagnosis of sleeping sickness in the blood stream.
- Venous or capillary blood is collected in a capillary tube until it is ¾ full, with anticoagulant. One end of the tube is sealed with clay and it is centrifuged at high speed for 5 minutes.
- Capillary tubes are then laid in a viewing chamber and examined under a microscope. Trypanosomes, if present, are seen as tiny moving organisms in the plasma, just above the layer of leukocytes (in the Buffy coat)

5.4 Sleeping sickness patient staging

- CATT dilution 1/16-positive, WOO Test-positive and Gland puncture-positive patients are all considered true cases of sleeping sickness, so "staging" is done on all of these patients to determine what stage of disease they are in.
- Staging of sleeping sickness patients is done by performing a lumbar puncture and analysis of cerebral spinal fluid.
- Treatment of patients is not begun without knowing the stage of the individual patient because the less toxic stage I drug is not effective in stage II, but all second stage drugs are extremely toxic; we do not want to put patients at risk by using the wrong drug.

6. Treatment of *T.b. gambiense*

6.1 Haemolymphatic stage (Stage I)

- **Pentamidine 200mg** intramuscular injection in buttocks/ gluteal muscle
  Dose 4 mg/ kg/ day for 7 days

6.2 Meningoencephalitic Stage (Stage II)

- DiFlorMethylOrnithine (DFMO, Eflornithine or ornidyl)
  Dose **adult** 100mg/kg 6 hourly infusion over 2 hour for 14 days
  Dose **paediatric** 150 mg /kg 6 hourly infusions over 2 hours for 14 days

6.3 Treatment of relapse or immune-compromised Stage II

- **Melarsoprol 180 mg**
  Dose 2.16 mg /kg i.v once a day for 10 days

7. Sleeping sickness & health workers in Magwi County

7.1 Sleeping sickness as a public health problem in Magwi County

- 1911: First time sleeping sickness known to be in South Sudan (1914 in Nimule area)
- 1920s – 1950s: Mobile screening of populations for SS and tsetse habitat clearance reduced SS to low levels in Magwi County
- 1960s – 1980s: Sleeping sickness probably present at low levels in the county, HAT found in refugees living in Opari, no control activities
- 1990s: War forced large populations to move in and out of Magwi County. Sleeping sickness moved with IDPs and soldiers coming from CES and WES
- 2000s: Returnees carried sleeping sickness with them from northern Uganda
- Present: Sleeping sickness has re-established itself in the human and tsetse fly population of Magwi County. **Sleeping sickness is again an important public health problem that health workers in this county must address.**

Figure: areas endemic for sleeping sickness in South Sudan and neighbouring countries

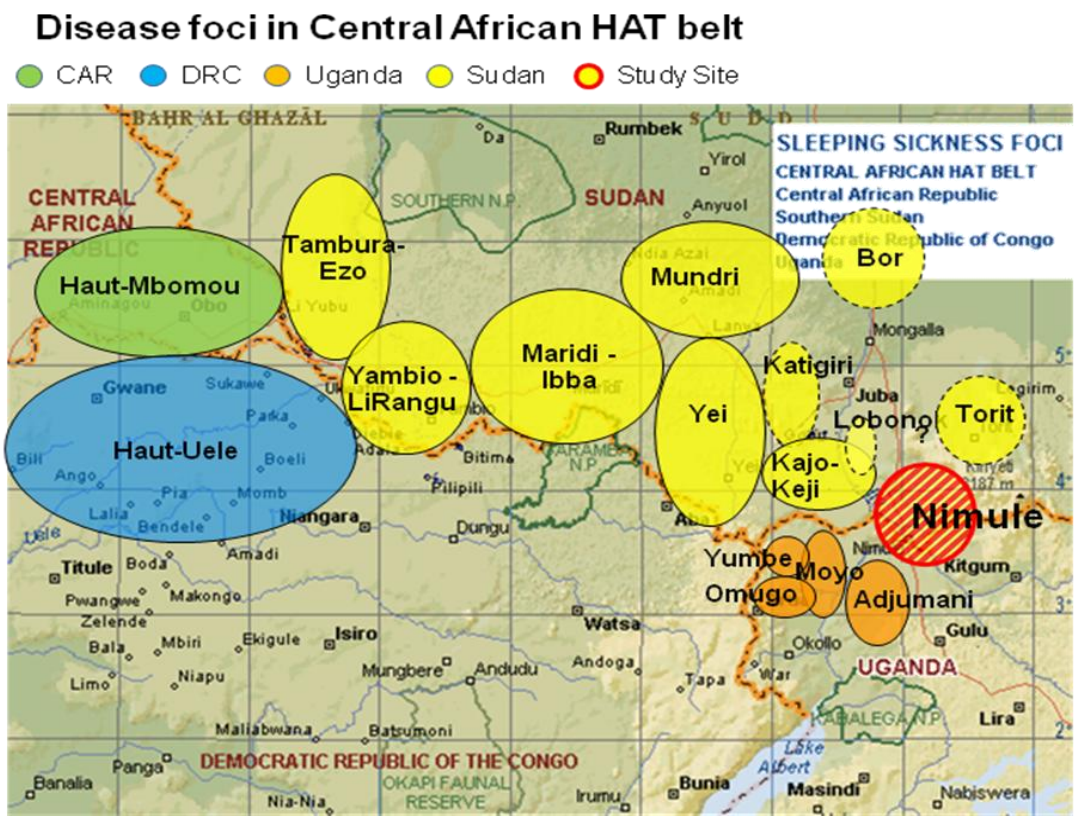

7.2 Mobile screening campaigns for sleeping sickness in Magwi County

Merlin conducted mobile screening surveys in 2005, 2006 and 2008 to try to identify cases in the early stage of disease and to reach patients who had poor access to testing facilities at the hospital. Over 15,000 people from 4 payams were screened in these surveys, and in those areas, approximately 1% of the population (1/100 people tested) were found to have SS. In some villages, the prevalence of SS was found to be higher than 5%. The prevalence in Lobone and Parajok payams is unknown.

Figure: HAT prevalence in Magwi County

Payam	Year	No. People screened	HAT prevalence (%)
Nimule	2005, 2006, 2008	5,756	1.0
Mugali	2006, 2008	2,945	1.4
Pageri	2005, 2006, 2008	5,166	1.0
Magwi	2006, 2008	1,851	0.7
Total		15,718	1.0

In all, 163 SS patients were detected during these surveys. Another 469 cases were detected from around 19,000 people screened passively at Nimule hospital during this period.

7.3 The role of health workers in sleeping sickness control

The advantage of using mobile screening teams to detect patients is that more patients are identified in stage I, so the disease is easier to control and patients are easier to treat. However, mobile teams cannot reach everyone in the county and may only come to individual villages once or twice a year. Also, governments and NGOs running SS control programs may not always have the funding to conduct this type of screening with mobile teams even if it is needed.

We found almost 3x more patients through passive screening at the hospital in the years we conducted screening surveys and health worker referrals are an important part of this passive screening system.

Therefore, **health workers should not rely on mobile teams to control SS in Magwi County.**

Sleeping sickness is a difficult disease for patients to diagnose in themselves because:

- Symptoms of sleeping sickness resemble other more common diseases such as malaria, typhoid, HIV and mental illnesses
- Sleeping sickness symptoms can also be mistaken for social phenomenon such as drunkenness, “over-thinking” or even evil spirits
- Sleeping sickness patients often try many different health facilities and different types of health workers for help with their symptoms, but many patients will eventually “give up” seeking help if no one suggests they go for a sleeping sickness test

- Very poor SS patients may give-up looking for help too early because they have other problems like lack of food, lack of money for transport
- Patients with psychiatric symptoms need extra help to recognize that they are sick and get to hospital
- Patients who drink a lot also need extra help to recognize that they are sick and get to hospital

For all these reasons, **health workers at all facilities in the county must assist sleeping sickness patients to recognize their signs & symptoms and refer them for a test at the hospital in order to control sleeping sickness in this county**.

7.4 Current sleeping sickness testing patterns in Magwi County

The figure below summarizes the amounts of people tested for sleeping sickness at Nimule hospital and the cases detected from each payam in the county. What is important to note in this figure is that the majority of cases detected (80%) currently come from Nimule payam, because over 90% of people who are tested for sleeping sickness are from this payam.

Figure: Numbers of people passively screened for SS by payam, Jan 2008 – Oct 2009

Payam	SS Testers	Proportion of testers	Cases	Proportion of cases
Nimule	2555	92%	92	80%
Mugali	60	2%	3	3%
Pageri	34	1%	6	5%
Magwi	18	0.6%	5	4%
Parajok	2	<0.1%	0	0
Lobone	28	1%	1	0.8%
Outside Magwi County	92	3%	11	9%
Total	2789		118	

From mobile screening surveys, we know that the prevalence of sleeping sickness is similar in each of the payams screened (around 1% in each payam) so this means that **the majority of sleeping sickness patient in areas outside of Nimule are currently not being recognised.**

7.5 Current referral practices for sleeping sickness tests

When sleeping sickness testers are categorized by the type of person who referred them for a test we can see that the majority of people who are tested are not sent by a health worker.

Figure: Referral source for sleeping sickness testers and cases, April – Oct 2009

Referral source	Testers	Cases
Health worker outside Nimule hospital	3	2
Nimule hospital inpatient ward	21	1
Nimule hospital OPD	23	3
Self / community member	719	22
Other	1	0
Total	767	28

In the last 6 months, only 3 people were referred for a sleeping sickness test by a health worker outside Nimule hospital. However, most patients that are detected at the hospital have already seen at least one health worker for help with their signs and symptoms of sleeping sickness. Therefore, **health workers in Magwi County have the opportunity to play a much larger role in identification of sleeping sickness patients in their payams**.

8. Taking effective patient histories

Sleeping sickness has many non-specific signs and symptoms and so they can mimic other more common diseases. As well, some of the signs are not recognized at first without an examination by a trained healthcare worker. It is therefore important to take full histories and physical examinations of all patients in order to determine the correct diagnosis. The following topics should be covered in your examinations:

i- Facility name
ii- Names
iii- Age
iv- Sex
v- Marital status
vi- Resident / village and payam
vii- Female parity
viii- Date
ix- Presenting complaints with their durations
x- History of present illness and symptoms
xi- Past medical history including any treatment given
xii- Drug allergies
xiii- Family history, ask whether there is chronic illness in family members e.g Tuberculosis, Asthma, Heart disease, Diabetes M , etc...
xiv- Social history, ask whether patient drinks alcohol or smokes, sexual behaviours
xv- Physical examinations:
General examination: weight, height, BMI , etc

Systemic examination: **inspection** from head to toe, **palpation**, **percussion** and **auscultation** of chest and abdomen when applicable

xvi- Differential Diagnosis

xvii- Investigations needed from the laboratory

xviii- Treatment / plus admission

9. Referral logs

9.1 Completing the sleeping sickness referral log

When you suspect one of your patients is suffering from sleeping sickness, please take this patient's history, perform an examination and record your findings in the sleeping sickness patient referral logs provided. Do this for all patients that you suspect may need a sleeping sickness test, and then send them to Nimule hospital for a test with a sleeping sickness referral slip.

Figure: Sleeping sickness patient referral log

Referral No. __1__ Date __1 November 2009__ Patient Name __Mary Acayo__

Sex __F__ Age __22__ Resident or IDP? __Res__ Village __Omeo__ Payam __Magwi__

Patient had SS before? (Y/N) __N__ Patient taken drugs for malaria or typhoid in last 2 weeks? (Y/N) __N__

Patient lives or works with (circle if yes): cows goats sheep pigs

Presenting signs & symptoms (circle if present):

Headache ≥1 week	Enlarged lymph nodes	Hallucinations
Back, neck or joint pain	Daytime sleeping	Convulsions
Muscle pain	Awake at night	Difficulty speaking
Fever ≥1 week	Confusion / forgetfulness	Difficulty walking
Itchy skin	Aggressiveness	Patient unsteady
Swollen face, legs or arms	Malaise / patient inactive	Jerking movements
Weight loss	Generally poor state of health	Tremor in hands or lips
Appetite: decrease	No menstruation	Partial paralysis
Appetite: increase	Impotence	Painful shin

Additional comments: __Patient also presents with cough, suspect ARI co-infection__

Referred by (health worker name) __James Omon__ Patient agreed to go for SS test? (Y/N) __Y__

After filling in the patient's personal information (name, age, address, etc), fill in information on 3 categories which may increase the likelihood that a patient has sleeping sickness:

- whether or not they have had sleeping sickness before,

- if they have already taken malaria or typhoid treatment in the last 2 weeks but their symptoms persist, and
- if they work with any animals that may also be infected with sleeping sickness or expose them to tsetse fly bites when taking animals to drink water from rivers.

Then record whether the patient has any signs or symptoms characteristic of sleeping sickness that you have learned from the patient history and examination you performed. In the comments section, record any other useful information such as other presenting symptoms or other diagnoses you are considering. If this patient was referred to you from a herbalist or witch doctor, please record this information here, as well.

Lastly, fill in your name and whether or not the patient agreed to go to Nimule hospital for a sleeping sickness test. Note: information should be recorded in these forms for all patients who you suspect might have sleeping sickness, regardless of whether or not they agree to go for a sleeping sickness test.

9.2 Completing the general referrals tally

Also remember to tally all patient referrals you make for any health problem, including for sleeping sickness testing and management. Mark a circle for every patient you refer, as on the form below, and start a new line for each week. Please separate the referrals you make according to whether you are referring to Nimule hospital or somewhere else.

Eg. If during the week of November 9th to 15th you made 5 referrals from your PHCU: 1 to Nimule hospital and 4 to Magwi PHCC, then your general referrals tally sheet should look like this:

Figure: General referrals tally

Month	Week	All referrals made to Nimule Hospital	Total	Referrals made to any other facility	Total
Nov	9-15	ØOOOO OOOOO OOOOO OOOOO	1	ØØØØO OOOOO OOOOO OOOOO	4
	16-22	OOOOO OOOOO OOOOO OOOOO		OOOOO OOOOO OOOOO OOOOO	

If you already keep a register of all referrals from your facility, continue with your usual record keeping system, and simply fill in this tally and the sleeping sickness referral log as well, for the duration of the study period.

If you do not normally keep a register of referrals from your facility, you may consider starting one. You can use a plain notebook and write in headings for every new page, for example, using the following format:

Figure: Setting up a general referral register

Name	Age	Sex	Village	Presenting symptoms	Diagnosis	Treatment	Comment
Mary Acayo	22	F	Omeo	Excessive sleeping, fever, headache, confusion, insomnia, cough	SS + ARI	Amoxil, paracetamol	Suspected SS patient referred for further management

10. Evaluation day in March 2010

We will return to each payam in March to evaluate the effectiveness of this health worker training programme on improving referrals for sleeping sickness tests at Nimule hospital. Please fill in the sleeping sickness referral log and general referrals tally for the entire study period from training (November/December 2009) to evaluation (March 2010).

On March 1st, 2010, please submit both of these records to the nearest

- **PHCU,**
- **Hospital, or**
- **Military barracks**

We do not yet know the exact dates for evaluations, but we will spend 2-3 days in each payam in this month going to PHCUs and barracks to collect these forms. We will hold group discussions with health workers who are available on those days to talk about their experiences identifying and referring patients.

As well, if there are any patients who were referred for a sleeping sickness test but did not go to the hospital and receive one, we will also interview them and their family members to learn about problems they may have had in getting a test.
