# Supplementary material for: A Mixed Methods Study of a Health Worker Training Intervention to Increase Syndromic Referral for Gambiense Human African Trypanosomiasis in South Sudan
Source: PLoS Negl Trop Dis. 2014 Mar 20;8(3):e2742. doi: 10.1371/journal.pntd.0002742 (PMC3961197; doi:10.1371/journal.pntd.0002742)
Supplement: File S2 — Syndromic HAT training HCW questionnaire. Pre-intervention questionnaire completed by all HCWs who attended training, including HAT symptom knowledge association test. (PDF) [file pntd.0002742.s002.pdf]

Participant No. _____

Magwi County health worker questionnaire

Personal Information

Q 1. The payam where I work is: (circle)

Nimule *Pageri* *Mugali* *Magwi* *Parajok* *Lobone*

Q 2. The type of place I work is: (circle)

Public health centre/unit/hospital *Private clinic*

Military medical corps *Traditional herbalist or witch doctor*

Q 3. Name of health facility: ____

Q 4. My job title is: ____

Q 5. My age is: (circle) *0-19 years* *20-29 years* *30-39 years*
40-49 years *50-59 years* *60 years or older*

Q 6. I am: (circle) *Male* *Female*

Q. 7 I can read and write: (circle) *Yes* *No*

Q 8. My nationality is: (circle) *Sudanese* *Ugandan* *Other* ___________

Q 9. My tribe is: (circle) *Madi* *Acholi* *Dinka* *Other* ___________

Q 10. How long have you been working as a health worker in Magwi County? ___ years ___ months

Health services information

Q 11. Are most (more than 70%) of your patients from one tribe? *Yes* *No*

If yes, which tribe are most of your patients from? *Madi* *Acholi* *Dinka* *Other*_____

Q 12. How many patients do **you, personally,** see on a typical day? (circle)

0-5 *6-10* *11-15* *16-20* *21 or more*

Q 13. Are you able to do any tests for patients at your facility? (circle) *Yes* *No*

Q 14. If yes, what kinds of tests are you able to do? (circle)

Malaria-RDT *Malaria-microscope* *Typhoid* *Syphilis*

Sleeping sickness-CATT   *Sleeping sickness-microscope*   *HIV*   *Tuberculosis*

Q 15. How often are these tests or reagents available to use? (circle)

Usually   *Sometimes*   *Not very often*

Referral practices

Q 16. Do you ever refer patients to another healthcare worker or facility? (circle)   *Yes*   *No*

Q 17. Where or who do you normally refer patients to? ___________________________________

Q 18. What are the most common conditions you refer patients for?

1______________________________   2_______________________________

3______________________________   4_______________________________

5______________________________

Q 19. What is the most common reason you refer patients? (circle one answer)

Emergency cases I can't treat myself

Non-emergency cases I can't treat myself

Cases I can normally treat, but can't at that moment because I don't have the drugs

Cases in which I don't know what is causing the problem

Other reason ____

Q 20. When was the last time you referred a patient? (approximate date) _____________________

Q 21. Why did you refer that patient? ____

____

Q 22. How many patients did you refer yesterday? (circle)   *0*   *1-3*   *4-10*   *11 or more*

Q 23. How many patients have you referred in the last week? (circle)

0   *1-3*   *4-10*   *11 or more*

Q 24. Do you currently keep any records on referrals made by you or your facility?   *Yes*   *No*

Sleeping sickness

Q 25. Have you ever seen someone with sleeping sickness before?   *Yes*   *No*

Q 26. Since you have been working in Magwi County, have you ever suspected sleeping sickness in one of your patients before?   *Yes*   *No*

Q 27. If yes, did you refer them for a sleeping sickness test?   *Yes*   *No*

Q 28. How many patients have you referred for a sleeping sickness test in the last month?

(circle) *0* *1* *2* *3* *4* *5 or more*

Q 29. Where do you normally refer for a sleeping sickness test? ________________________

Q 30. In your opinion, how common a problem are the following illnesses in Magwi County: (circle)

Malaria?	*Not common*	*Somewhat common*	*Very common*
Typhoid?	*Not common*	*Somewhat common*	*Very common*
Syphilis?	*Not common*	*Somewhat common*	*Very common*
HIV?	*Not common*	*Somewhat common*	*Very common*
Tuberculosis?	*Not common*	*Somewhat common*	*Very common*
Sleeping sickness?	*Not common*	*Somewhat common*	*Very common*
Mental illness?	*Not common*	*Somewhat common*	*Very common*
Drunkenness?	*Not common*	*Somewhat common*	*Very common*
Poisoning?	*Not common*	*Somewhat common*	*Very common*

Q 31. Of these problems just listed, which do you feel are the three most important problems in this area?

1.____________________ 2. ______________________ 3.______________________

Q 32. Is this a sign or symptom of sleeping sickness?

Fever for 2 days	*Yes*	*No*	*I don't know*
Fever for a week or more	*Yes*	*No*	*I don't know*
Severe headache	*Yes*	*No*	*I don't know*
Cough	*Yes*	*No*	*I don't know*
Itching skin	*Yes*	*No*	*I don't know*
Daytime sleeping	*Yes*	*No*	*I don't know*
Loss of appetite	*Yes*	*No*	*I don't know*
Increase in appetite	*Yes*	*No*	*I don't know*
Abdominal pains	*Yes*	*No*	*I don't know*
Difficulty walking	*Yes*	*No*	*I don't know*
Infertility	*Yes*	*No*	*I don't know*
Convulsions	*Yes*	*No*	*I don't know*
Not wanting to talk to others	*Yes*	*No*	*I don't know*
Seeing or hearing things that are not there	*Yes*	*No*	*I don't know*

Q 33. What do you hope to get out of this workshop? ____

____
